# Precision obesity medicine: A phenotype-guided framework for pharmacologic therapy across the lifespan

**DOI:** 10.1007/s40618-025-02700-7

**Published:** 2025-11-10

**Authors:** Dario Tuccinardi, Davide Masi, Mikiko Watanabe, Valeria Zanghi Buffi, Francesco De Domenico, Sabrina Berti, Valentina Cipriani, Melania Manco, Silvia Manfrini, Uberto Pagotto

**Affiliations:** 1 Department of Endocrinology and Diabetes, University Campus Bio-Medico of Rome, Rome, 00128 Italy; 2 Fondazione Policlinico Universitario Campus Bio-Medico, 00128, Rome, Italy; 3 Department of Experimental Medicine, Section of Medical Pathophysiology, Food Science and Endocrinology, Sapienza University of Rome, Rome, 00161 Italy; 4 Department of Clinical and Surgical Sciences, University of Bologna, Bologna, 40126 Italy; 5 Division of Endocrinology and Diabetes Prevention and Care, Department of Medical and Surgical Sciences, IRCCS Azienda Ospedaliero-Universitaria di Bologna, Alma Mater Studiorum University of Bologna, Bologna, Italy; 6 Research Unit for Preventive and Predictive Medicine, Bambino Gesù Children’s Hospital, IRCCS, Rome, Italy

**Keywords:** Personalized obesity medicine, Anti-obesity pharmacotherapy, Phenotype-guided treatment, Incretin-based therapies, Precision medicine in obesity

## Abstract

**Objective:**

Obesity is a biologically complex and heterogeneous disease that requires individualized, phenotype- and complication-oriented therapeutic strategies. The introduction of advanced pharmacotherapies, including GLP-1 receptor agonists (GLP-1 RA), dual Glucose-dependent Insulinotropic Polypeptide/Glucagon-like Peptide-1 (GIP/GLP-1) agonists, and emerging triple agonists, has facilitated a shift from weight-centric goals to precision-based obesity care. This review provides a clinical framework for pharmacologic treatment, organized by phenotype, obesity-related complications, age, and behavioral traits.

**Design:**

Narrative review of randomized trials, meta-analyses, real-world evidence, and international guidelines through May 2025. Evidence was synthesized across key obesity phenotypes, cardiometabolic, hepatic, renal, mechanical, behavioral, and stratified by life stage, including pediatric, reproductive-age, and older adults, with attention to safety, cost-effectiveness, and special populations.

**Results:**

In established Atherosclerotic Cardiovascular Disease, semaglutide significantly reduces major adverse cardiovascular events. Tirzepatide offers cardiometabolic benefits for high-risk people without overt disease. Both agents improve symptoms and function in Heart Failure with Preserved Ejection Fraction, irrespective of glycemia or weight loss. In Chronic Kidney Disease, they decrease albuminuria and eGFR decline. In Metabolic Dysfunction-Associated Steatotic Liver Disease, GLP-1 RAs and GIP/GLP-1 RAs demonstrate marked histological improvements. Mechanical complications such as osteoarthritis and sleep apnea are improved by anti-obesity medications-induced weight loss. GLP-1 RAs and naltrexone/bupropion prove effective against binge and emotional eating. In youths, liraglutide and semaglutide are both approved and effective. Liraglutide and orlistat preserve lean mass alongside resistance training and adequate protein intake in older and sarcopenic people.

**Conclusions:**

An anti-obesity treatment framework focused on both phenotype and complication burden improves the personalization of obesity care and supports clinical decision-making throughout a person’s lifespan.

**Supplementary Information:**

The online version contains supplementary material available at 10.1007/s40618-025-02700-7.

## Introduction

 Obesity is now firmly recognized as a complex, chronic, relapsing, and progressive disease, affecting over 650 million adults worldwide. It is a major driver of type 2 diabetes (T2D), atherosclerotic cardiovascular disease (ASCVD), heart failure, chronic kidney disease (CKD), metabolic dysfunction-associated steatotic liver disease (MASLD), and a broad spectrum of mechanical and neurodevelopmental and neurobehavioral complications [1]. Historically approached with uniform weight-centric strategies, obesity care is now transitioning towards a precision medicine model that accounts for its biological heterogeneity and multisystemic impact.

While intentional weight loss confers benefit across virtually all obesity-related conditions, the magnitude and nature of clinical improvement depend not only on the degree of weight reduction, but also on the specific mechanism of action of the intervention and the underlying phenotype. The optimal therapeutic strategy, be it lifestyle intervention, pharmacotherapy, or metabolic surgery, should therefore be personalized according to three critical axes: (1) the strength of evidence linking the intervention to improvement in the target complication; (2) the weight loss threshold required for disease modification; and (3) the presence of pleiotropic, weight-independent effects.

If obesity-related complications are already present, pharmacologic treatment should be considered as first-line, in combination with lifestyle therapy, without the need to delay until lifestyle interventions alone are attempted.

This model has already transformed T2D care, where antihyperglycemic therapies are selected not only for their glucose-lowering efficacy but for their ability to reduce cardiovascular events, preserve renal function, and improve hepatic or heart failure outcomes. A parallel paradigm shift is now occurring in obesity medicine. Incretin-based pharmacotherapies, including GLP-1 receptor agonists (GLP-1 RAs), such as semaglutide, and dual GIP/GLP-1 agonists like tirzepatide, as well as emerging triple agonists, achieve Weight reductions of 15–20% or more in randomized controlled trials. They also demonstrate significant improvements in glycemic control, blood pressure, hepatic steatosis, cardiovascular outcomes, and quality of life [2–5].

As understanding of the disease has deepened, it has become clear that obesity comprises multiple pathophysiological and behavioral phenotypes. These include insulin-resistant obesity, lipotoxic hepatic obesity, sarcopenic obesity, and eating behavior subtypes such as binge eating disorder and emotional eating [6]. Phenotypic stratification allows for more rational, effective, and safe use of anti-obesity medications (AOMs). For instance, GLP-1 RAs may be preferred in people with emotional eating due to their satiety-enhancing effects. At the same time, naltrexone/bupropion may be more effective in cases with strong hedonic drive or reward dysregulation [7]. Similarly, older adults with sarcopenic obesity may benefit from agents that help preserve lean mass or combine safely with resistance training [7].

Early pharmacologic intervention in people without overt complications may prevent progression to more advanced disease. For example, semaglutide and tirzepatide in prediabetes have shown remarkable efficacy in restoring normoglycemia and preventing T2D onset. In people with obesity and high cardiovascular risk, the initiation of AOMs can reduce major adverse cardiovascular events (MACE) before the development of frank ASCVD [8]. This preventative potential underscores the shift from a reactive to a proactive therapeutic stance, mirroring other chronic disease models such as hypertension and dyslipidemia.

With expanding evidence from clinical trials and real-world studies, including data across the life course from adolescence to late adulthood, there is an urgent need for a structured and clinically pragmatic framework to guide treatment selection. Such a framework should incorporate phenotypic characterization, encompassing metabolic, hepatic, renal, cardiovascular, mechanical, behavioral, and age-related dimensions, and align drug mechanisms with therapeutic objectives.

This narrative review integrates evidence from 1998 to 2025 to support phenotype-based pharmacologic strategies for obesity. It aims to provide clinicians with a comprehensive and actionable guide to precision obesity medicine. The primary objective is to link scientific evidence with clinical practice, ensuring that treatment choices are customized to weight targets and the broader aim of preventing complications, modifying disease, and enhancing patient-centered outcomes. Importantly, this review does not propose a hierarchical approach, starting with lifestyle, followed by drugs, and ultimately surgery, as a universal treatment algorithm. Instead, it aims to map the available evidence that informs phenotype-based pharmacologic decision-making. Noteworthy, only pharmacotherapy currently EMA and/or FDA approved will be discussed. Given the frequent overlap among clinical phenotypes, the choice of therapy should arise from a shared decision-making process between clinician and patient, prioritizing the most clinically relevant complication and the most meaningful one to the individual. Therapeutic goals, expected benefits, tolerability, contraindications, and cost considerations must all be assessed to ensure a sustainable and personalized approach to care.

## Methods

A multidisciplinary panel of endocrinologists, pediatricians, registered dietitians and obesity medicine specialists conducted a structured narrative review of the literature from January 1998 to May 2025 using a PRISMA process. The aim was to identify and synthesize clinical trial evidence and expert consensus documents supporting phenotype-guided pharmacologic treatment of obesity across various age groups and comorbid conditions.

A comprehensive literature search was conducted between January 1998 and May 2025 using PubMed/MEDLINE, Scopus, Cochrane CENTRAL, Clinicaltrials.gov, regulatory sources (EMA, FDA), and manual searches of high-impact journals (*NEJM*,* Lancet*,* JAMA*,* Diabetes Care*,* Obesity*,* Nature Medicine*,* Cell Metabolism*). The search terms (combined with Boolean operators) used were “obesity” AND (“phenotype” OR “precision medicine” OR “endotype”) AND (“pharmacotherapy” OR “GLP- 1 receptor agonist” OR “tirzepatide” OR “semaglutide” OR “liraglutide”, OR “phentermine topiramate“ OR “naltrexone bupropion” OR “orlistat” OR “anti- obesity medication” OR “bariatric surgery”) AND (“RCT” OR “trial” OR “real- world evidence”) AND (“children” OR “older adult” OR “T2D” OR “HFpEF” OR “CKD” OR “MASLD” OR “OSA” OR “sarcopenia” OR “binge eating” OR “emotional eating” OR “PCOS”). The inclusion criteria were randomized controlled trials, meta-analyses, observational studies, and regulatory reviews published in English; trials reporting phenotypic or subgroup analyses on pharmacologic obesity treatments; articles discussing phenotype-based treatment algorithms or precision obesity strategies; and relevant expert consensus statements or clinical guidelines. The exclusion criteria included animal studies, narrative editorials, non-peer-reviewed publications, and articles without abstract or full-text access.

A total of 1.296 titles and abstracts were initially screened. After applying the inclusion/exclusion criteria, 212 full texts were reviewed. Ultimately, 200 studies were included in the final synthesis, comprising randomized controlled trials (RCTs), meta-analyses, observational studies and guideline documents. Studies were grouped by phenotypic domains (e.g., T2D, CKD, HFpEF, MASLD, OSA, bariatric surgery, sarcopenia, monogenic obesity and syndromic obesities). Extracted data included: population characteristics, intervention type/dose, phenotype stratification, weight loss magnitude, and complication-specific outcomes. No quantitative meta-analysis was performed. Instead, data were narratively synthesized and organized by clinical phenotype and life stage.

Phrases such as *“should be preferred*,*” “may be considered*,*”* and *“not recommended”* were systematically assigned to each pharmacologic option across phenotypic domains. These phrases reflect the strength of evidence, regulatory status, and safety profile, as agreed upon by the authors after review of the available data.

“Should be preferred”: Applied when at least one Phase III randomized controlled trial with prespecified, phenotype-specific primary endpoints demonstrated clinically meaningful benefit, with regulatory approval and no major safety concerns.

“May be considered”: Applied when evidence was derived from Phase II trials, Phase III trials with secondary or post-hoc endpoints, consistent real-world data, or surrogate outcomes plausibly linked to disease modification, in the absence of definitive outcomes.

“Not recommended”: Applied when efficacy was not demonstrated, or when relevant safety issues, contraindications, or an unfavorable risk–benefit profile were present.

Detailed “Clinical Takeaways” boxes are available in the Supplementary Material. They provide clear, practice-oriented summaries of the recommendations for each phenotype, enabling rapid translation of the evidence into clinical decision-making.

## Cardio renal metabolic complication-based pharmacotherapy

### Atheerotic cardiovascular disease (ASCVD) and heart failure (HF)roscl

**Fig. 1:**
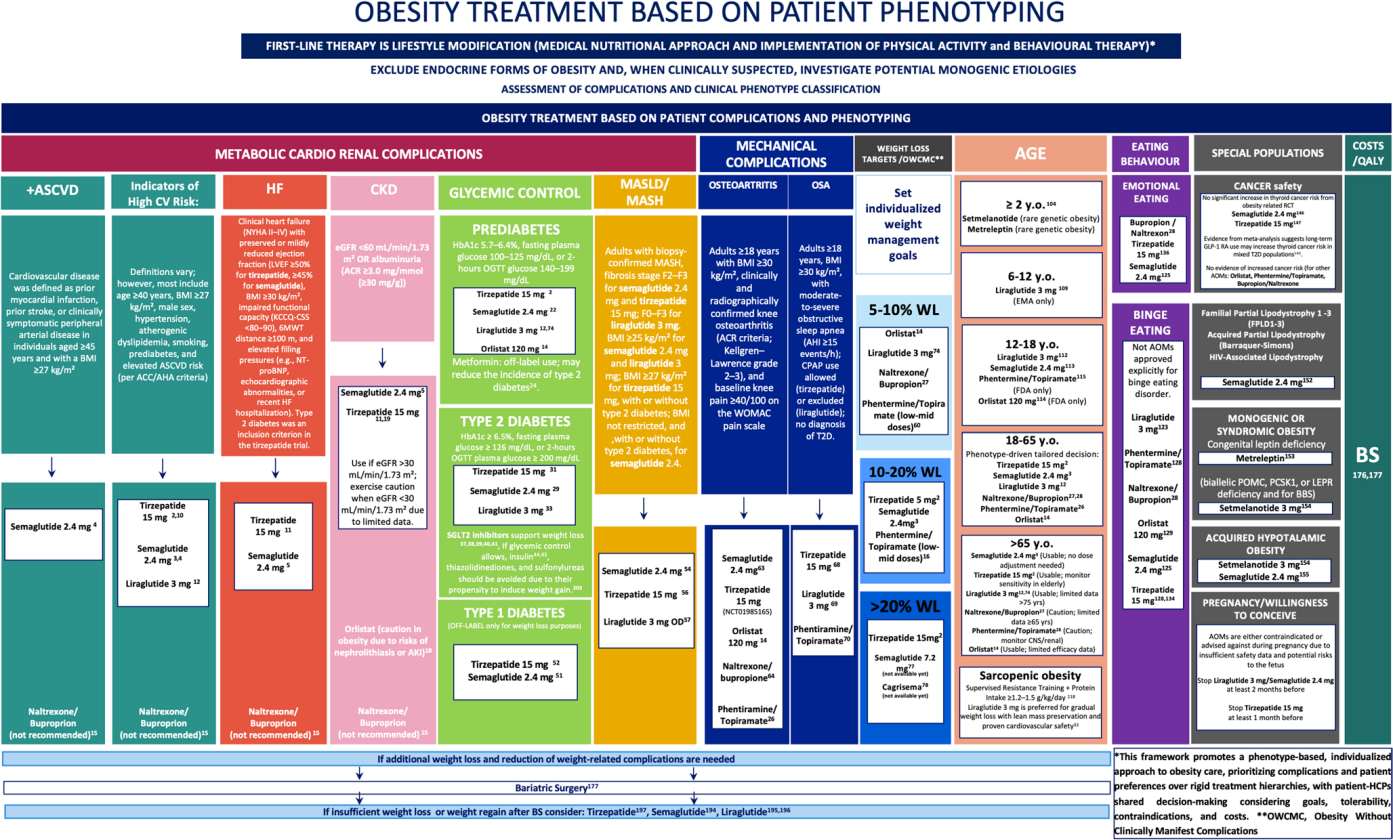
A Phenotype-Guided Framework for Obesity Pharmacotherapy.This figure presents a structured, phenotype-based model for pharmacologic obesity treatment, in which each column corresponds to a major complication domain of obesity. Within each column, content is organized in a hierarchical sequence: first, the clinical complication (e.g., type 2 diabetes, ASCVD, MASLD, CKD, OSA, OA, eating disorders); second, the diagnostic definitions and inclusion criteria used in pivotal trials that demonstrated efficacy of anti-obesity medications (AOMs) in that phenotype; third, the pharmacologic agents tested in those populations, ordered from top to bottom according to the strength of recommendation, beginning with agents that should be prioritized based on evidence and magnitude of effect. Where applicable, the figure also outlines referral to bariatric surgery when pharmacologic strategies are insufficient, followed by the option of re-introducing AOMs after surgical failure or weight regain. This sequence underscores the need for dynamic treatment strategies based on response rather than rigid therapeutic escalation. Rather than endorsing a fixed stepwise progression from lifestyle to drugs to surgery, the framework supports a personalized approach integrating patient phenotype, complication burden, treatment goals, and shared decision-making. The model reflects a shift toward complication-driven precision medicine in obesity, emphasizing early, targeted intervention and long-term outcome optimization. Abbreviations: AOMs, Anti-Obesity Medications; ASCVD, Atherosclerotic Cardiovascular Disease; HFpEF, Heart Failure with Preserved Ejection Fraction; CKD, Chronic Kidney Disease; MASLD, Metabolic Dysfunction-Associated Steatotic Liver Disease; MASH, Metabolic Dysfunction-Associated Steatohepatitis; T2DM, Type 2 Diabetes Mellitus; T1DM, Type 1 Diabetes Mellitus; PCOS, Polycystic Ovary Syndrome; OSA, Obstructive Sleep Apnea; OA, Osteoarthritis; OWCMC, Obesity Without Clinically Manifest Complications; EBD, Eating Behavior Disorder; AHI, Apnea–Hypopnea Index; CPAP, Continuous Positive Airway Pressure; KCCQ-CSS, Kansas City Cardiomyopathy Questionnaire Clinical Summary Score; eGFR, Estimated Glomerular Filtration Rate; ACR, Albumin-to-Creatinine Ratio; NYHA, New York Heart Association; LVEF, Left Ventricular Ejection Fraction; QALY, Quality-Adjusted Life Year; BS, Bariatric Surgery; GI, Gastrointestinal

**Fig. 2:**
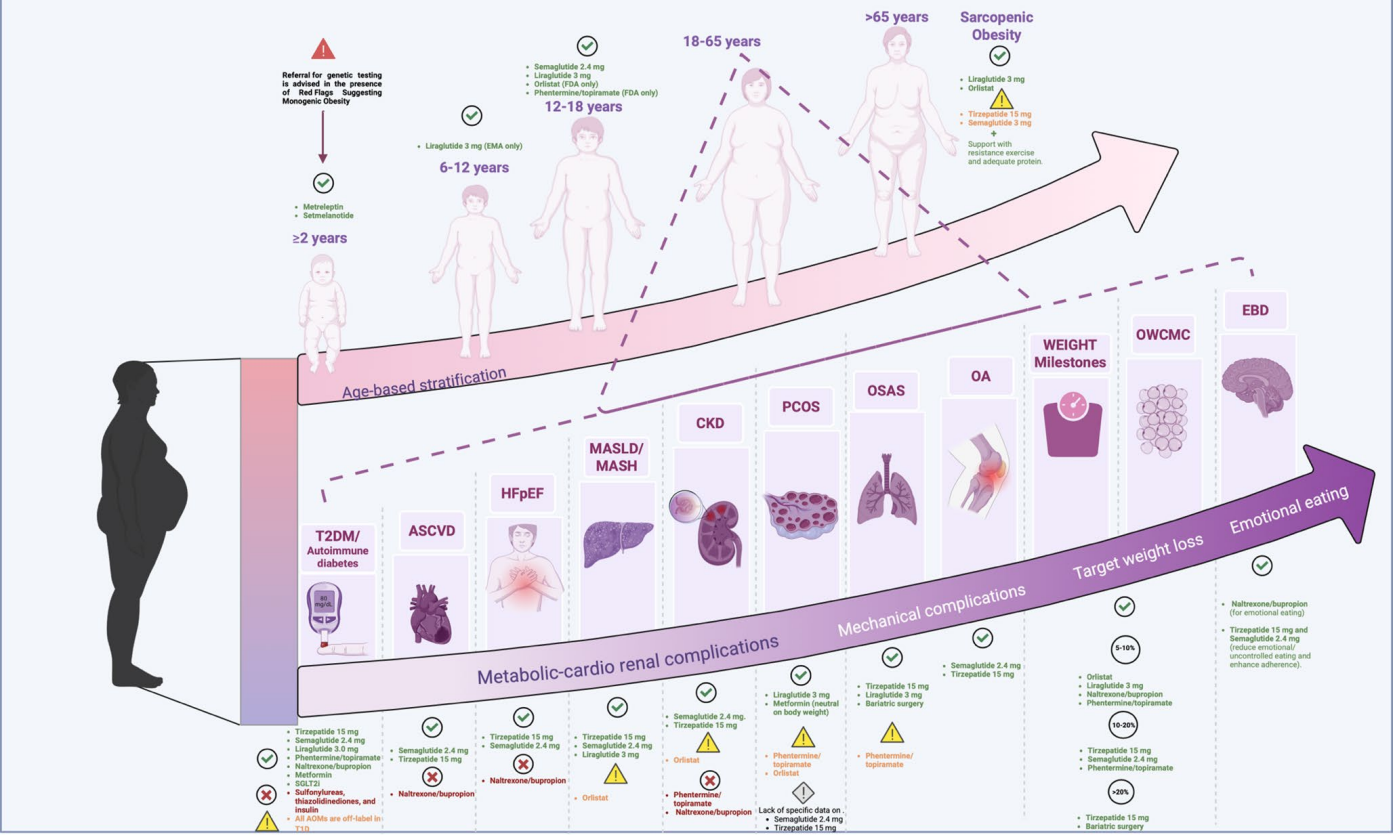
Precision Obesity Medicine: Integrating Age, Phenotype, and Pharmacotherapy.This figure illustrates a comprehensive framework for precision obesity care, integrating age-based stratification (upper trajectory), phenotype-driven treatment selection (lower trajectory), and weight loss targets across the lifespan. The top section delineates recommended pharmacologic options according to developmental stage—from infancy (≥ 2 years) to sarcopenic obesity in late adulthood—highlighting specific agents with supporting evidence and safety considerations. The lower section maps obesity-related complications along a phenotypic continuum, spanning metabolic–cardio–renal diseases (e.g., type 2 diabetes, ASCVD, CKD, MASLD/MASH), mechanical complications (e.g., osteoarthritis, OSA), and behavioral phenotypes (e.g., emotional eating), together with target weight loss milestones (5%, 10–20%, > 20%). Each condition is annotated with preferred pharmacologic agents, based on available efficacy and safety data. Agents are color-coded by recommendation grade (green = preferred; yellow = caution or off-label; red = contraindicated). The figure supports individualized, complication-oriented obesity management and underscores the need for phenotype-guided drug selection beyond a weight-centric model. BMI, Body Mass Index; T2DM, Type 2 Diabetes Mellitus; ASCVD, Atherosclerotic Cardiovascular Disease; HFpEF, Heart Failure with Preserved Ejection Fraction; MASLD, Metabolic Dysfunction-Associated Steatotic Liver Disease; MASH, Metabolic Dysfunction-Associated Steatohepatitis; CKD, Chronic Kidney Disease; PCOS, Polycystic Ovary Syndrome; OSAS, Obstructive Sleep Apnea Syndrome; OA, Osteoarthritis; OWCMC, Obesity Without Clinically Manifest Complications; EBD, Eating Behavior Disorder

Cardiovascular disease remains the leading cause of death in people with obesity, even in the absence of diabetes. Obesity contributes to ASCVD and HF via metabolic, inflammatory, and hemodynamic mechanisms. Visceral and epicardial fat deposition drives insulin resistance, endothelial dysfunction, and neurohormonal activation. Heart Failure with Preserved Ejection Fraction (HFpEF), in particular, has emerged as an obesity-mediated phenotype characterized by impaired compliance, elevated filling pressures, and limited exercise tolerance. As discussed by Tuccinardi et al., AOMs targeting adiposity and CV risk offer a novel treatment paradigm, mirroring recent T2D strategies [9] (Figs. 1 and 2; Tables 1 and 6) (see Supplementary Material for Clinical Takeaways).

**Table 1:** Cardiovascular, heart failure, and renal outcomes of pharmacologic interventions for obesity

Intervention	ASCVD benefit	HFpEF benefit	Evidence quality	Clinical recommendation	CVD evidence source	Renal benefit	Evidence quality	Clinical recommendation	CKD evidence source
Semaglutide 2.4 mg	↓ MACE 20%, ↓ all-cause mortality 19%	↓ HF events, ↑ QoL, ↑ 6MWD	ASCVD: High	ASCVD: Preferred	[4, 5]	↓ eGFR decline, ↓ macroalbuminuria, ↓ ESRD risk	Moderate	Preferred	[17]
			HFpEF:Moderate	HFpEF: symptom and functional improvement, appropriate alternative to tirzepatide					
Tirzepatide 15 mg	No MACE data, post hoc studies suggest ↓ ASCVD risk	↓ HF hospitalization by 46%, ↑ QoL, ↑ 6MWD	ASCVD:Moderate	ASCVD: Promising for cardiovascular risk reduction. CVOT pending for ASCVD.	[10, 11]	↑ eGFR (creatinine and cystatin C), ↓ albuminuria	Moderate	Promising option; renal-specific outcome trials needed	[11, 19]
			HFpEF: High	HFpEF: Preferred					
Liraglutide 3.0 mg	No MACE data, post hoc studies suggest no ASCVD increase	No HF data	ASCVD + HFpEF:Low	ASCVD: No recommendation; HFpEF: No recommendation	[13, 74]				
Orlistat 120 mg	Neutral MACE	No HF data	ASCVD + HFpEF:Low	ASCVD: Consider when preferred treatments contraindicated; HFpEF: No recommendation	[14, 202]	↓ CKD progression, ↔ eGFR	Low	Consider ifpreferredtreatmentscontraindicated; monitor for oxalate nephropathy	Observational cohort; nonrandomised CKD trial
Naltrexone/Bupropion 8/90 mg	No MACE data	No HF data	ASCVD + HFpEF:Low	ASCVD/HFpEF: Not recommended	[15]	No renal data	Low	Notrecommended	General obesity trials; no CKD subgroups
Phentermine/Topiramate 15/92 mg	No MACE data	No HF data	ASCVD + HFpEF:Low	ASCVD/HFpEF: Not recommended	[16]	No renal benefit	Low	Notrecommended in moderate-to-severe CKD	[16]

This table summarizes the effects of pharmacologic and surgical interventions for obesity on three major complication domains: atherosclerotic cardiovascular disease (ASCVD), heart failure with preserved ejection fraction (HFpEF), and chronic kidney disease (CKD). Arrows indicate direction of effect: ↓ denotes improvement (e.g., ↓ MACE = reduction in major adverse cardiovascular events), ↑ denotes increase (e.g., ↑ QoL = improved quality of life), and ↔ indicates no significant change. Evidence quality (high, moderate, or low) reflects the strength of supporting data: high denotes large randomized trials or meta-analyses with predefined endpoints; moderate, post hoc analyses or secondary endpoints from RCTs; and low, observational or non-randomized data. Clinical recommendations are based on integration of benefit, safety, and quality of evidence. Trial sources are specified in the rightmost columns. All data refer to people with obesity and without diabetes, unless otherwise stated. Abbreviations: ASCVD, atherosclerotic cardiovascular disease; HFpEF, heart failure with preserved ejection fraction; CKD, chronic kidney disease; MACE, major adverse cardiovascular events; QoL, quality of life; 6MWD, 6-minute walk distance; eGFR, estimated glomerular filtration rate; ESRD, end-stage renal disease; CVOT, cardiovascular outcomes trial; RCT, randomized controlled trial; CVD, cardiovascular disease

**Semaglutide**

In the SELECT trial, semaglutide 2.4 mg once weekly (OW) reduced MACE (CV death, nonfatal myocardial infarction (MI), stroke) by 20% (HR 0.80; 95% CI 0.72–0.90) and all-cause mortality by 19% in subjects with obesity and established ASCVD but without diabetes [4]. In STEP-HFpEF, semaglutide 2.4 OW improved Kansas City Cardiomyopathy Questionnaire clinical summary score (KCCQ-CSS) (+ 7.8 points), 6-minute walk distance (6MWD) (+ 20.3 m), reduced C Reactive Protein (CRP) (−43.5% vs. −7.3%) and body weight (− 13.3% vs. − 2.6%), and showed a lower, though exploratory, HF event rate (HR 0.08; 95% CI 0.00–0.42) [5]. Semaglutide **should be preferred** as first-line AOM in people with obesity and established ASCVD or HFpEF, even in the absence of diabetes, based on robust CV benefit.

**Tirzepatide**

Though CVOT data are pending, post hoc SURMOUNT-1 analyses showed reduced 10-year ASCVD risk and improved blood pressure, lipids, and inflammation in participants without diabetes [10]. In the SUMMIT trial, tirzepatide significantly lowered the composite of CV death or HF hospitalization (HR 0.62; 95% CI 0.41–0.95), driven by reduced HF hospitalizations (HR 0.54; 95% 0.34–0.85), with no significant effect on CV death. It also improved KCCQ-CSS (+ 6.9 points, placebo subtracted) and 6MWD (18.3 m; 95% CI, 9.9–26.7) [11]. Tirzepatide **should be preferred** for people with obesity and HFpEF; the CV (MACE) benefit in obesity appears promising but awaits dedicated CVOT data SURMOUNT MMO Trial, ClinicalTrials.gov (ID NCT05556512).

**Liraglutide**

In a 3-year trial in 2254 participants with obesity and prediabetes, liraglutide 3.0 mg improved CV risk factors including blood pressure and lipids [12]. No data support liraglutide for HF prevention in obesity without diabetes. In a post hoc analysis from SCALE randomized controlled trials found that liraglutide 3.0 mg was not associated with increased cardiovascular risk compared to the comparator groups (placebo or orlistat). However, the results are limited by a small number of events and retrospective adjudication [13]. Liraglutide **may be considered** in people with obesity and elevated CV risk who are not candidates for more potent agents; not explicitly recommended for HF.

**Orlistat**

The XENDOS trial reported modest weight loss and BP reduction, with a neutral cardiovascular profile [14]. Orlistat **may be considered** when centrally acting AOMs are contraindicated, though long-term efficacy is limited.

**Naltrexone/Bupropion**

The LIGHT trial was terminated early; the impact of MACE remains unknown [15]. Sympathomimetic effects (↑HR, BP) limit its use in ASCVD or HF. EMA recommends annual cardiovascular evaluation due to possible long-term risks. This combination is **not recommended** for people with ASCVD or HF due to potential hemodynamic stress and lack of outcome data, especially when alternatives with proven benefits are available.

**Phentermine/Topiramate**

No CVOTs exist. Though effective for weight loss, phentermine’s sympathomimetic profile (↑HR, BP) necessitates caution in high-risk people. EQUIP showed modest BP and lipid improvement, but cardiovascular safety is uncertain [16]. Noteworthy, the combination Phentermine/Topiramate is FDA- but not EMA-approved. It is therefore not a viable option in Europe. This combination **is not recommended** for high-risk people with ASCVD or HF, especially when alternatives with proven benefit are available.

### Chronic kidney disease (CKD)

CKD is a common and serious complication of obesity, amplifying cardiovascular risk, impairing quality of life, and limiting therapeutic options. Its pathophysiology involves glomerular hyperfiltration, intrarenal inflammation, and ectopic fat accumulation in the renal sinus and perirenal space, exacerbated by hypertension, insulin resistance, and dyslipidemia. Even modest renal impairment alters drug clearance and increases adverse effect risk, making AOM use in CKD a high-stakes clinical decision (Figs. 1 and 2; Tables 1 and 6) (See Supplementary Material for Clinical Takeaways).

**Semaglutide**

In the SELECT trial, semaglutide 2.4 mg reduced a composite renal endpoint (≥ 50% eGFR decline, macroalbuminuria onset, or need for renal replacement therapy) by 22% vs. placebo [17]. This prespecified endpoint occurred alongside major adverse CV events and all-cause mortality reductions, suggesting a comprehensive cardio-renal-metabolic effect. Semaglutide up to 1 mg OW was found to be safe in dialysis patients with obesity in a prospective observational study, but its use remains not permitted in clinical practice for this population [18]. Semaglutide **should be preferred** as first-line AOM in people with obesity and CKD, including non-diabetic populations, based on renal and CV outcome data.

**Tirzepatide**

Although not yet assessed in a dedicated renal outcome trial, tirzepatide has shown consistent renal benefits across subgroups. In SUMMIT (HFpEF + obesity), tirzepatide improved creatinine–and cystatin C–based eGFR and reduced UACR, though these were secondary and not prespecified endpoints [11]. SURMOUNT-1 post hoc analyses confirmed favorable shifts in renal biomarkers in participants with obesity and no diabetes [19]. Tirzepatide **may be considered** when semaglutide is not tolerated, although renal benefits remain to be confirmed in dedicated trials (TREASURE-CKD, ClinicalTrials.gov ID NCT05536804).

**Orlistat**

As a non-systemic lipase inhibitor, orlistat may be considered in CKD where systemic agents are unsuitable. A small study of patients with stage 3–5 CKD facilitated Weight loss and preserved renal function over 2 years, improving transplant eligibility [20]. A population study showed reduced risk of incident CKD stage ≥ 3 (HR 0.78), CV events, and mortality [21]. However, oxalate nephropathy remains a concern, particularly in advanced CKD, requiring close monitoring. Orlistat **may be considered** in people with moderate-to-severe CKD when incretin therapies are contraindicated, with close renal monitoring.

**Naltrexone/Bupropion**

No renal-specific data are available. Its sympathomimetic effects (↑BP, HR) raise concern for renal harm in moderate-to-severe CKD. Without outcome data and given the potential hemodynamic burden, this combination should be avoided in CKD. This combination **is not recommended** to people with moderate-to-severe CKD (eGFR < 60 mL/min/1.73 m²), given uncertain benefit and safety concerns.

**Phentermine/Topiramate ER**

The EQUIP trial (15) showed efficacy in weight loss and cardiometabolic risk factors, but renal outcomes were not evaluated. Phentermine’s sympathomimetic activity and topiramate’s partial renal clearance raise concerns, especially in reduced eGFR, due to risks of acidosis and nephrolithiasis. This combination is not recommended in moderate-to-severe CKD. This combination **is not recommended** to people with eGFR < 60 or with nephrolithiasis risk, due to potential for harm and lack of renal outcome data.

### Prediabetes

Prediabetes, defined by impaired fasting glucose, impaired glucose tolerance, or elevated HbA1c, represents a critical window for metabolic intervention in people with obesity. Excess adiposity drives insulin resistance, β-cell dysfunction, and hepatic steatosis, contributing to progressive dysglycemia. AOMs that induce meaningful weight loss can restore normoglycemia and reduce diabetes risk. (Figures 1 and 2) (See Supplementary Material for Clinical Takeaways).

**Semaglutide**

In STEP 1, semaglutide 2.4 mg OW led to 10–15% Weight loss and restoration of normoglycemia in 84.1% of prediabetic participants over 68 weeks [3]. Improvements in HOmeostatic Model Assessment for Insulin Resistance (HOMA-IR) index, fasting glucose, and HbA1c further support its use as a first-line agent in obesity-related prediabetes.

In STEP 10, OW semaglutide 2.4 mg resulted in significant Weight loss of approximately 12% over 68 weeks compared to placebo in people with obesity and prediabetes. Normoglycemia was restored in a substantially higher proportion of participants receiving semaglutide versus placebo. Improvements in fasting glucose, HbA1c, and insulin sensitivity measures further validate semaglutide’s efficacy and safety as a treatment option for obesity-associated prediabetes [22]. Semaglutide **should be preferred** in people with obesity and prediabetes, based on high rates of diabetes prevention and metabolic improvement.

**Tirzepatide**

In SURMOUNT-1, tirzepatide 15 mg OW achieved 22.5% mean Weight loss and a 94% risk reduction in diabetes progression over 72 weeks in participants with prediabetes [2]. Post hoc analyses confirmed insulin sensitivity and glycemic marker improvements [10]. In a specially designed trial for Obesity Treatment and Diabetes Prevention involving people with overweight or obesity and prediabetes, tirzepatide caused significant, dose-dependent weight loss (− 12.3% with 5 mg, − 18.7% with 10 mg, and − 19.7% with 15 mg compared to − 1.3% with placebo; *P* < 0.001) and significantly lowered the incidence of T2D (1.3% vs. 13.3%; HR 0.07; *P* < 0.001) [23]. Tirzepatide **should be preferred** in people with obesity and prediabetes, when greater weight loss is needed.

**Metformin**

The Diabetes Prevention Program showed that metformin 850 mg BID reduced T2D incidence by 31% over 2.8 years, with greater benefits for younger people with higher Body Mass Index (BMI) [24]. While cost-effective and well-tolerated, its modest weight effect (~ 2–3 kg) limits its utility when weight loss is a primary goal. It is not specifically approved for prediabetes. Metformin **may be considered** in prediabetes, especially when cost or tolerability is a concern; less effective for weight-driven glycemic reversal.

**Acarbose** Used adjunctively, acarbose modestly lowers weight ~ 0.5 kg (difference 0·77 kg [95% CI 0·01–1·54] compared to placebo) by delaying carbohydrate absorption and promoting satiety [25]. Acarbose **may be considered adjunctively** in select people with postprandial hyperglycemia, though limited by GI side effects and minimal weight benefit.

**Orlistat**

In XENDOS, orlistat reduced T2D incidence by 45% over four years [14], with a ~ 2.9 kg greater weight loss versus placebo and modest insulin sensitivity gains. Due to dietary fat restrictions, GI side effects and adherence challenges may limit its broader use. Orlistat **may be considered** when incretin-based therapies are contraindicated, particularly in early-stage dysglycemia.

**Phentermine/Topiramate ER**

In a post hoc CONQUER analysis, phentermine/topiramate ER reduced T2D progression by 48–78% over 56 weeks, depending on dose and baseline glycemia, driven by up to 10.9% weight loss and improved insulin sensitivity [26]. This combination **may be considered** in prediabetes requiring more potent weight loss, pending psychiatric and CV risk profile.

**Naltrexone/Bupropion**

Although not formally tested in T2D prevention trials, data from COR-I [27] and COR-II [28] showed reductions in fasting glucose and HOMA-IR. This agent **may be considered** in people with obesity, emotional eating, and mild dysglycemia without significant CV risk.

### Type 2 diabetes (T2D)

Obesity and T2D share common mechanisms, insulin resistance, chronic low-grade inflammation, ectopic lipid accumulation, and progressive β-cell failure. Over 80% of people with T2D suffer from overweight or obesity. Weight loss of ≥ 5–15% is associated with meaningful reductions in HbA1c, insulin needs, and cardiometabolic complications. AOMs now play a central role in managing both weight and glucose in this population (Figs. 1 and 2; Tables 2 and 6) (see Supplementary Material for Clinical Takeaways).

**Table 2:** Weight loss, glycemic efficacy, and evidence appraisal of Anti-Obesity medications in type 2 diabetes

Intervention	Weight loss	HbA1c reduction	Evidence quality	Clinical recommendation	Evidence source
Semaglutide 2.4 mg	9.6%	−1.6%	High	Recommended agent especially if secondary CVD prevention	[29]
Tirzepatide 15 mg	14.7%	−2.08%	High	Recommended agent for dual hypoglycemic and weight efficacy in T2D	[31]
Liraglutide 3.0 mg	6.0%	–1.3%	High	Alternative for daily use; proven CV protection at 1.8 mg	[33]
Orlistat 120 mg	2.9–3.4%	−0.3% to − 0.5%	Moderate	Consider when systemic agents are contraindicated	[14]
Naltrexone/Bupropion 8/90 mg	∼5.0%	−0.6%	Moderate	Useful in selected patients with appetite dysregulation and mild hyperglycemia	[35]
Phentermine/Topiramate 15/92 mg	−9.4%	−0.4%	Moderate	Consider in selected patients prioritizing weight loss	[36]

This table summarizes the effects of anti-obesity pharmacotherapies on body weight and HbA1c in people with type 2 diabetes (T2D). Weight loss and glycemic outcomes are expressed as approximate ranges from pivotal trials and representative real-world studies. Evidence quality is categorized as high or moderate, based on study design and outcome consistency: high denotes large randomized trials or meta-analyses with predefined endpoints; moderate refers to RCTs with secondary endpoints, real-world studies, or population subanalyses. Clinical recommendations reflect integrated assessment of efficacy, cardiovascular benefit, route of administration, and patient phenotype. All agents are considered in combination with lifestyle intervention and glucose-lowering background therapy as needed. Abbreviations: T2D, type 2 diabetes; CVD, cardiovascular disease; HbA1c, glycated hemoglobin; RWE, real-world evidence; RCT, randomized controlled trial

**Semaglutide**

In STEP 2, semaglutide 2.4 mg led to 9.6% mean weight loss in people with T2D, as expected, slightly less than in non-diabetic populations; HbA1c reductions of ~ 1.6% and improved insulin sensitivity were observed [29].

**It should be preferred** in people with T2D and obesity who require combined Weight and glycemic control. Based on the MACE reduction observed in SUSTAIN 6 [30], **it should be preferred** in patients with established ASCVD (secondary prevention).

**Tirzepatide**

Tirzepatide outperforms other AOMs in glycemic and weight outcomes. In SURPASS-1 to −5, Weight loss ranged from 11 to 15%. SURMOUNT-2 (obesity + T2D) confirmed 14.7% mean Weight loss at 15 mg over 72 weeks [31]. HbA1c reduction − 2.08%, with 84% of participants achieving HbA1c < 7.0%, and 49% HbA1c < 5.7%. In the SURPASS 2 trial, tirzepatide 15 mg action improves insulin secretion, reduces glucagon and hepatic glucose output, and shows better glycemic control than the semaglutide 1 mg OW [32]. **It should be preferred** in people with T2D and obesity when both HbA1c and weight burden are high, or when semaglutide is insufficient. It currently offers the most potent dual effect on glycemia and weight.

**Liraglutide**

Liraglutide 3.0 mg achieved ~ 6.0% Weight loss and 1.3% Hba1c reductions in the SCALE Diabetes trial [33]. At 1.8 mg, it reduced MACE by 13% (primary outcome) and CV mortality by 22% in LEADER [34]. It **may be considered** in patients with ASCVD or when daily injection is preferred; a valid alternative if weekly agents are not tolerated.

**Non-Incretin AOMs in T2D**

**Orlistat**

Orlistat achieved 2.9–3.4% greater weight loss and modest HbA1c reductions (0.3–0.5%) vs. placebo, mostly via improved insulin sensitivity [14]. GI side effects and modest efficacy limit its utility. It **may be considered** in people unable to use systemic AOMs, but adherence and efficacy are limited.

**Naltrexone/Bupropion**

In COR-Diabetes, naltrexone/bupropion yielded − 5% weight loss and − 0.6% HbA1c reductions [35]. It **may be considered** in those needing potent weight loss without CV/psychiatric contraindications.

**Phentermine/Topiramate ER**

In a pooled subgroup analysis including T2D subjects of the CONQUER and the OB-202/DM-230 Study, the 15/92 mg dose reduced HbA1c by 0.4% (placebo subtracted) and led to up to 9.4% Weight loss over 56 weeks [36]. It is helpful in patients where weight loss is the primary goal. However, sympathomimetic effects limit its use in uncontrolled hypertension or ASCVD. It m**ay be considered** in selected people with emotional eating and controlled BP/psychiatric history.

#### Effect of Non-Incretin-Based antidiabetic medications on body weight

Weight control is central to T2D management, influencing glycemic control, cardiovascular risk, and long-term adherence. Antidiabetic agents vary in their weight effects, which should guide personalized therapeutic choices. Agents that support weight control **should be preferred** over those with a neutral or promoting weight effect in people living with T2D and obesity, particularly SGLT2i in those suffering from HF and/or diabetic kidney disease (Figs. 1 and 2; Table 3) (see Supplementary Material for Clinical Takeaways).

**Agents Supporting Weight Control**

**Metformin**

Long considered first-line therapy, metformin is associated with modest weight loss or weight stability. In the Diabetes Prevention Program, metformin led to a mean weight reduction of ~ 2.1 kg (1.8–4.6 kg) over 2.8 years [24].

**Table 3:** Weight effects, glycemic efficacy, and clinical role of Non-Incretin-Based antidiabetic agents

	Effect on weight	HbA1c reduction	Evidence quality	Clinical recommendation	Evidence source
Metformin	↓ ∼2 kg	−1.0% to − 1.5%	High	First-line therapy in T2D with weight-neutral to mild weight benefit	[24]
Acarbose	↓ ∼1–2 kg (1.32 ± 2.37)	−0.1%	Moderate	Adjunctive option in postprandial hyperglycemia; mild weight benefit	[37]
SGLT2i (High effect: Canagliflozin 300 mg, Ertugliflozin 15 mg)	↓ 3–5 kg	−0.5% to − 1.0%	High	Preferred among SGLT2i when weight loss is a major goal	[38, 39]
SGLT2i (Moderate effect: Empagliflozin, Dapagliflozin, Empaglifozin)	↓ 2–3 kg	−0.5% to − 1.0%	High	Commonly used; good balance of efficacy and safety in T2D with CV benefit	[40, 41]
SGLT2i (Lower effect: Sotagliflozin)	∼1.5–2 kg	−0.5% to − 0.8%	Moderate	May be considered when dual SGLT1/2 inhibition is indicated	[42]
DPP-4 Inhibitors	↔ Neutral	−0.5% to − 0.8%	Moderate	Useful in elderly or renal impairment when weight neutrality is desired	[43]
Sulfonylureas	↑ Gain (∼2–3 kg)	−1.0% to − 1.5%	Moderate	Use with caution due to hypoglycemia risk and weight gain	[203]
Thiazolidinediones	↑ Gain (up to 4 kg)	−0.5% to − 1.4%	Moderate	Consider in NAFLD or insulin resistance; monitor for fluid retention	[45]
Insulin	↑ Gain 1–2 Kg, although variable)	−1.0% to − 2.0%	High	Essential if beta-cell failure; combine with weight-sparing agents if needed	[46]

This table presents the effects of non-incretin-based antidiabetic medications on body weight and HbA1c levels in people with type 2 diabetes (T2D). Weight impact is expressed as approximate average change in kilograms or categorized as neutral or gain. Arrows indicate direction of effect: ↓ denotes weight loss, ↑ indicates weight gain, and ↔ indicates no significant change. Evidence quality is graded as high or moderate based on randomized controlled trials (RCTs), meta-analyses, and real-world effectiveness. Clinical recommendations reflect appropriate use based on phenotype, comorbidity profile, and drug tolerability. Medications are organized by class and stratified by weight effect, from weight loss to weight gain. Abbreviations: T2D, type 2 diabetes; SGLT2i, sodium–glucose cotransporter 2 inhibitor; SGLT1/2, sodium–glucose cotransporter 1 and 2; DPP-4, dipeptidyl peptidase 4; NAFLD, non-alcoholic fatty liver disease; CV, cardiovascular; HbA1c, glycated hemoglobin; RCT, randomized controlled trial

**Acarbose**

A slight weight reduction was observed over the study period (1.32 ± 2.37 kg for acarbose versus 0.43 ± 2.9 kg for placebo patients), but the difference was not statistically significant (*P* = 0.13) [37].

**SGLT2 Inhibitors**

These agents reduce body weight via glycosuria-induced caloric loss, with class-specific and agent-specific heterogeneity in the magnitude of weight reduction.

*SGLT2is with Higher Weight Reduction (3–5 kg)*:

Ertugliflozin induces clinically meaningful weight loss in people with T2D, reducing 4.12 kg in those with class I obesity and 6.82 kg in those with baseline BMI > 35 kg/m² [38].

Canagliflozin 300 mg led to a 3.6 kg weight loss (4.2%) over 104 weeks in type 2 diabetes patients on metformin [39].

*SGLT2is with Moderate Weight Reduction (~ 2–3 kg)*:

Empagliflozin 10 and 25 mg: Over 24 weeks, empagliflozin 10 mg and 25 mg reduced body Weight by 2.0 kg and 2.4 kg, respectively, compared to 0.4 kg with placebo [40].

Dapagliflozin 10 mg: Dapagliflozin alone caused a 2.7 kg Weight loss after 52 weeks, while combined with exenatide, loss increased to 3.8 kg [41].

*SGLT2is with Lower Weight Reduction (~ 1.5–2 kg)*:

Sotagliflozin, a dual SGLT1/2 inhibitor, leads to modest weight loss (~ 1.3 kg), which is generally lower than that observed with selective SGLT2 inhibitors, with greater interindividual variability due to its dual intestinal and renal mechanism of action [42].

**Agents with a weight-neutral effect**

**DPP-4 Inhibitors**

Consistently weight-neutral. A pooled sitagliptin analysis confirmed no significant weight change [43].

**Agents Associated with Weight Gain**

**Sulfonylureas**

Increase insulin secretion regardless of glycemia, typically resulting in 2–3 kg weight gain [44].

**Thiazolidinediones (TZDs)**

Due to fluid retention and fat redistribution, rosiglitazone and pioglitazone are linked to 3–4 kg weight gain [45].

**Insulin**

Exogenous insulin promotes lipogenesis and suppresses lipolysis, especially with intensive control. ORIGIN reported a 1.6 kg gain over 6 years with insulin glargine [46]. When basal insulin is required, a combination with GLP1RAs **should be preferred** over basal insulin alone in people with T2D and obesity [47].

### Autoimmune diabetes

Obesity is increasingly common in people with Autoimmune diabetes, complicating glycemic control and metabolic health. As noted by Maddaloni et al., this “double diabetes” phenotype combines autoimmune β-cell failure with insulin resistance and visceral adiposity [48]. Excess fat mass in Autoimmune diabetes heightens insulin requirements and contributes to hepatic steatosis, systemic inflammation, and microvascular and skeletal complications. Exogenous insulin may worsen weight gain and glycemic variability, fueling a cycle of dysmetabolism [49].

AOMs and bariatric surgery are emerging tools to reduce metabolic overload and insulin demand, even without β-cell recovery (Figs. 1 and 2, Supplementary Table 1) (see Supplementary Material for Clinical Takeaways).

**Semaglutide**

In a real-world cohort of 50 adults with T1D and overweight/obesity, semaglutide use over 12 months led to significant weight loss and HbA1c improvement, without increased hypoglycemia risk [50]. Combined with automated insulin delivery, Semaglutide resulted in an average weight loss of − 8.8 kg (95% CI, − 10.6 to −7.0). Each group experienced two severe hypoglycemia events, and no cases of diabetic ketoacidosis were reported. These effects probably indicate appetite suppression and insulin regulation sensitization [51]. It **may be considered** in selected people with T1D and obesity to reduce insulin requirement and body weight, with appropriate monitoring.

**Tirzepatide**

In a retrospective study of 51 T1D people with obesity, tirzepatide was associated with 8.5% mean Weight loss, 0.9% HbA1c reduction, and a 31.6% decrease in daily insulin dose over 8 months, with no increase in hypoglycemia; GI symptoms were the main side effect; overall tolerability was high [52].

Semaglutide and tirzepatide demonstrated significant weight loss and improved glycemic control in overweight and obese adults with T1D over 12 months. Semaglutide users experienced an average Weight loss of 9.1% (approximately 8.7 kg), while tirzepatide users lost 21.4% of their body weight (approximately 49.4 lbs). Both groups also showed reductions in HbA1c: a 0.54% decrease for semaglutide and a 0.68% decrease for tirzepatide, with statistical significance (*P* < 0.0001) [53].

**Tirzepatide may be considered** in selected patients with T1D, marked obesity and insulin resistance, though its off-label use requires close glycemic monitoring.

### Metabolic dysfunction-associated steatotic liver disease (MASLD/MASH)

MASLD (formerly NAFLD) reflects the hepatic manifestation of systemic metabolic dysfunction. Its progressive subtype, MASH (previously NASH), is defined histologically by ballooning, lobular inflammation, and fibrosis. Obesity is the principal driver, promoting insulin resistance, visceral adiposity, dyslipidemia, and inflammation.

Sustained weight loss ≥ 10% is the cornerstone of MASH treatment but rarely achieved through lifestyle alone. AOMs capable of inducing substantial weight loss have emerged as disease-modifying agents. Incretin-based therapies are currently the most promising for histologic resolution, fibrosis regression, and transaminase normalization (Figs. 1 and 2; Tables 4 and 6) (see Supplementary Material for Clinical Takeaways).

**Table 4:** Resolution of steatohepatitis and fibrosis improvement with Anti-Obesity therapies in MASLD/MASH

Intervention	MASH resolution	Fibrosis improvement	Evidence quality	Clinical recommendation	Evidence source
Semaglutide 2.4 mg	62.9%	36.80%	High	Recommended (off-label) in MASH w/or w/o fibrosis	[55]
Tirzepatide 15 mg	44–62%	51–55%	Moderate	Recommended (off-label) in MASH w/or w/o fibrosis	[56]
Liraglutide 3 mg	39%	Reduced progression	Moderate	May be used for early MASH in selected patients	[57]
Orlistat 120 mg	Not different than control	Not different than control	Low	May improve metabolic and liver enzyme parameters in MASLD but is not recommended as a first-line treatment for MASLD or MASH	[58]
Naltrexone/Bupropion 8/90 mg	Not assessed	Not assessed	Very Low	No recommendation	[59]
Phentermine/Topiramate 15/92 mg	Not assessed	not assessed	Low	No recommendation	[60]

This table summarizes the impact of anti-obesity pharmacotherapies on histologic endpoints in metabolic dysfunction-associated steatohepatitis (MASH), with or without hepatic fibrosis. MASH resolution and fibrosis improvement are reported as percentages from liver biopsy–controlled trials or characterized as “not different from control” or “not assessed” where applicable. Evidence quality is graded as high when derived from large, randomized, placebo-controlled trials with histological endpoints as primary outcomes; moderate for smaller RCTs or post hoc analyses with biopsy-based data; low for studies with non-significant effects or limited histologic data; and very low for studies lacking direct liver outcome assessment. Clinical recommendations reflect expert opinion on off-label use, based on the balance between efficacy, safety, and evidence strength in patients with MASLD or biopsy-confirmed MASH. Abbreviations: MASLD, metabolic dysfunction-associated steatotic liver disease; MASH, metabolic dysfunction-associated steatohepatitis

**Semaglutide**

In a 72-week phase 2 RCT of 1197 participants with biopsy-confirmed MASH and F2–F3 fibrosis, semaglutide 0.4 mg/day achieved a significant MASH resolution in 59% vs. 17% with placebo [54]. Fibrosis improvement (43% vs. 33%) did not reach significance, likely due to study duration. Semaglutide also reduced liver fat, which was assessed using magnetic resonance imaging-derived proton density fat fraction (MRI-PDFF), transaminases, and non-invasive fibrosis markers. Though not yet approved, off-label use is widespread, especially in people with T2D or metabolic Syndrome. In the Phase 3 ESSENCE trial, semaglutide 2.4 mg OW significantly improved liver histology in 800 patients with MASH and moderate-to-advanced fibrosis over 72 weeks. Resolution of steatohepatitis without worsening fibrosis occurred in 62.9% of semaglutide-treated patients compared to 34.3% in the placebo group (*P* < 0.001). Additionally, 36.8% of the semaglutide group experienced reduced liver fibrosis without worsening steatohepatitis, versus 22.4% in the placebo group (*P* < 0.001). The combined endpoint of both outcomes was achieved in 32.7% of the semaglutide group compared to 16.1% in the placebo group (*P* < 0.001) [55]. Semaglutide **should be preferred** in people with overweight/obesity and biopsy-proven MASH or MASLD with fibrosis and concurrent T2D or metabolic syndrome. Although not yet approved, off-label use is shared and supported by robust RCT data.

**Tirzepatide**

In a 52-week phase 2b trial in 157 participants with biopsy-proven MASH, tirzepatide significantly increased resolution of MASH without worsening of fibrosis compared to placebo: 44% with 5 mg, 56% with 10 mg, and 62% with 15 mg versus 10% in the placebo group (*P* < 0.001 for all doses). Fibrosis improvement without MASH worsening was also more frequent with tirzepatide (51–55% vs. 30% with placebo), with gastrointestinal events being the most common adverse effects, generally mild to moderate [56]. Tirzepatide **may be considered** in people with MASH and high weight burden or insulin resistance, especially when semaglutide is not tolerated.

**Liraglutide**

The LEAN study showed 39% of patients on liraglutide 1.8 mg achieved MASH resolution without fibrosis worsening, compared to 9% with placebo [57]. Liraglutide also reduced fibrosis progression and improved liver enzymes and hepatic insulin sensitivity. Liraglutide **may be considered** in early MASLD or if higher-potency incretins are not tolerated. Effect less robust in advanced fibrosis.

**Orlistat**

Although biopsy-controlled data are lacking, orlistat improves steatosis, ALT, and insulin sensitivity in MASLD when paired with lifestyle changes. A meta-analysis confirmed modest reductions in liver fat and enzymes but did not affect liver fibrosis. Benefits were not significant in NASH patients [58]. Weight loss (~ 3–4%) is modest, and GI side effects may limit adherence. Orlistat **may be considered** in early MASLD when incretin-based therapies are contraindicated; monitor for GI intolerance.

**Naltrexone/Bupropion**

Evidence in MASLD is limited and indirect. Some studies suggest modest reductions in hepatic fat and transaminases, but no histologic data support use [59]. It **may be considered** in MASLD/MASH; may be cautiously considered in patients with mild steatosis and emotional eating, avoiding use in hepatic dysfunction.

**Phentermine/Topiramate ER**

In CONQUER and related analyses, this combination reduced ALT and improved insulin sensitivity. Weight loss ranges from 10 to 12%, aligning with MASH remission thresholds, though no biopsy-controlled trials exist [60]. This combination **may be considered** in patients with visceral obesity and MASLD when incretins are unsuitable, though it lacks histologic efficacy data.

## Mechanical complications of obesity

### Osteoarthritis (OA)

OA is a progressive degenerative joint disease marked by cartilage breakdown, bone remodeling, and chronic pain, especially in weight-bearing joints. Obesity contributes to OA through both biomechanical overload and metabolic inflammation. Adipose-derived cytokines (e.g., IL-6, TNF-α, leptin) promote chondrocyte catabolism and systemic inflammation, worsening joint degeneration [61].

Epidemiological data show that each 5-unit BMI increase raises knee OA risk by 35% [62]. Weight loss is thus a key intervention, with growing evidence for AOMs and bariatric surgery as disease-modifying approaches. (Figures 1 and 2) (See Supplementary Material for Clinical Takeaways).

**Semaglutide**

In the STEP 9 trial involving adults with obesity and knee osteoarthritis, semaglutide resulted in significantly greater weight loss (− 13.7% vs. −3.2%), pain reduction (WOMAC − 41.7 vs. −27.5 points), and improvement in physical function (SF-36 + 12.0 vs. +6.5 points) compared to placebo (all *P* < 0.001) at 68 weeks, with a similar rate of serious adverse events [63]. Semaglutide **should be preferred** in people with obesity and knee OA who meet criteria for pharmacologic weight management, particularly when pain and function impair quality of life.

**Tirzepatide**

No trial has yet demonstrated direct efficacy of tirzepatide in OA. However, the STOP KNEE-OA phase 4 trial (clinicaltrial.gov n NCT01985165) is underway to assess its impact on delaying knee replacement and improving joint outcomes. Tirzepatide achieves ~ 21% weight loss [2]a level associated with OA symptom relief in other contexts. Tirzepatide **may be considered** in patients with obesity and OA where greater weight loss is required, pending dedicated OA outcomes.

**Orlistat**

Orlistat has shown modest OA symptom improvement through > 10% weight loss [14]. GI side effects may limit adherence. Orlistat **may be considered** when other AOMs are contraindicated or not tolerated, especially in early OA, with adherence counseling.

**Naltrexone/Bupropion**

While effective for weight reduction, direct OA studies are lacking. Theoretical benefits exist via reduced inflammation and appetite control, but clinical confirmation is absent [64]. **Should not be routinely recommended** for OA symptom improvement; use only if indicated for other behavioral phenotypes and with caution.

**Phentermine/Topiramate ER**

This agent induces significant weight loss (10–12%) [26] which may alleviate OA symptoms. However, OA-specific data are lacking, and use remains extrapolative. Based on extrapolated benefits from weight reduction, this combination **may be considered** in patients with obesity and OA who are not candidates for incretin therapies.

### Obstructive sleep apnea

Obstructive Sleep Apnea (OSA) is a prevalent obesity-related complication marked by recurrent upper airway collapse during sleep, causing intermittent hypoxia, hypercapnia, and sleep disruption. It affects 40–60% of people with obesity, with prevalence increasing in parallel with BMI [65]. Visceral fat, pharyngeal adiposity, and reduced airway tone elevate airway collapsibility [66]. Shared mechanisms, leptin resistance, sympathetic activation, and systemic inflammation, link OSA to cardiometabolic risk [67]. OSA is associated with hypertension, arrhythmias, stroke, T2D, MASLD, and HFpEF, and independently increases cardiovascular and all-cause mortality. Integrating OSA management within obesity treatment frameworks is essential. Weight loss remains the cornerstone of OSA management, but pharmacologic agents that induce significant weight loss and potentially modulate central respiratory pathways may offer added benefit (Figs. 1 and 2) (see Supplementary Material for Clinical Takeaways).

**Tirzepatide**

Tirzepatide became the first drug approved by the FDA for moderate-to-severe OSA in adults with obesity in December 2024. In the phase 3 (SURMOUNT-OSA-1 and − 2), tirzepatide significantly reduced apnea–hypopnea index (AHI) over 52 weeks in adults with moderate-to-severe obstructive sleep apnea and obesity, both with and without background PAP therapy. Mean AHI reductions were − 25.3 and − 29.3 events/hour with tirzepatide vs. − 5.3 and − 5.5 with placebo (*P* < 0.001 for both trials). Specifically, tirzepatide 15 mg led to a 62.8% reduction in AHI vs. 6.4% with placebo over 52 weeks and alongside Weight loss averaged 18.1%, with concurrent improvements in oxygen saturation, daytime sleepiness, and blood pressure. Benefits were consistent across OSA severity and comorbidities [68]. Tirzepatide **should be preferred** in people with obesity and moderate-to-severe OSA, particularly when PAP therapy is poorly tolerated or insufficient.

**Liraglutide**

In the SCALE Sleep Apnea trial, liraglutide 3.0 mg reduced AHI by − 12.2 events/h vs. − 6.1 with placebo (*P* = 0.015) over 32 weeks, with 5.7% vs. 1.6% weight loss (*P* < 0.0001) [69]. Modest improvements in oxygenation and blood pressure were also observed. Liraglutide **ay be considered** in patients with obesity and mild-to-moderate OSA when tirzepatide is contraindicated or unavailable.

**Phentermine/Topiramate ER**

A 28-week RCT showed that mid/high-dose phentermine/topiramate in 45 subjects with moderate to severe OSA not on positive airway pressure (PAP) treatment significantly reduced AHI (− 31.5 vs. − 16.6 events/h with placebo; *P* = 0.0084), with improved oxygenation and sleep quality [70]. It **may be considered** in patients with OSA and obesity where rapid weight loss is indicated, and other agents are not suitable, but caution is warranted in patients with CV risk.

**Naltrexone/Bupropion and Orlistat**

These agents support weight loss but lack robust evidence for OSA-specific benefit. No high-quality trials have demonstrated consistent reductions in AHI; thus, their role remains secondary and indirect. It **may be considered**, however, their role is limited to indirect benefit via weight loss.

## Clinical weight loss milestones and therapeutic stratification

Weight reduction in people with obesity yields a continuum of clinical benefits, with specific thresholds linked to improvements in glycemia, cardiometabolic risk, liver disease, and quality of life. A weight loss of ≥ 5% represents the first clinically meaningful milestone, associated with improved metabolism, enhanced glucose metabolism and blood pressure. A 10–15% loss may induce remission of T2D or MASLD. While > 20% is typically required to reverse advanced metabolic or hepatic disease [71]. These effects were initially established with lifestyle and surgical interventions [72], but pharmacotherapy now offers benefits similar to those of additional pleiotropic actions. (Figures 1 and 2; Tables 5 and 6) (See Supplementary Material for Clinical Takeaways).

**Table 5:** Anti-Obesity pharmacotherapy: classification by weight loss magnitude and therapeutic profile

Expected weight loss	Intervention	Key clinical benefits	Evidence quality	Evidence source
3–5%	Orlistat 120 mg	↓ diabetes incidence	Moderate	[14]
	Naltrexone/Bupropion	↓ emotional/binge eating	Moderate	[27]
5–10%	Liraglutide 3.0 mg	↑ glucose control, ↓ T2D progression	High	[74]
	Phentermine/Topiramate (low–mod dose)	Glucose + liver marker improvement	Moderate	[60]
10–15%	Tirzepatide 5–10 mg	↑ glucose control, liver, CV, and metabolic benefits	High	[2]
	Semaglutide 2.4 mg	↑ glucose control, liver, CV, and metabolic benefits	High	[3]
	Phentermine/Topiramate (high dose)	↓T2D and prediabetes	Moderate	[16]
≥ 20%	Tirzepatide 15 mg	Tirzepatide 15 mg: ↑ glucose control, CV, and metabolic benefits, MASH improvement/resolution	High	[2]
	Semaglutide 7.2 mg	Not available on the market at the publication date Semaglutide 7.2 mg achieved weight loss (− 20.7%) in adults with obesity, with one in three participants losing ≥ 25% of body Weight. While tolerability was slightly reduced, safety remained acceptable, supporting dose escalation as a viable intensification strategy beyond 2.4 mg.	High	[76]
	CagriSema (coadministration of cagrilintide 2.4 mg and semaglutide 2.4 mg)	Not available on the market at the publication date coadministration of cagrilintide 2.4 mg and semaglutide 2.4 mg (CagriSema) resulted in a 20.4% mean Weight loss at 68 weeks in adults with overweight or obesity. Over half of participants achieved ≥ 20% weight loss, and nearly one in five lost ≥ 30% of body weight. While gastrointestinal adverse events were frequent (79.6%), they were mostly transient and mild to moderate, supporting CagriSema as a highly effective future option for weight management beyond current monotherapies	High	[78]

This table presents pharmacological interventions for obesity stratified by expected mean Weight loss categories and corresponding clinical benefits. Each intervention is listed with the key metabolic and clinical advantages observed in trials, along with the quality of supporting evidence and primary literature sources. Medications are grouped by average percentage of total body Weight loss observed in RCTs or extension studies: 3–5%, 5–10%, 10–15%, and ≥ 20%. For interventions not yet approved at the time of publication (e.g., Semaglutide 7.2 mg and CagriSema), pivotal phase 3 trial data are included to contextualize potential future indications. Evidence quality is categorized as high, moderate, or low, based on the design of the supporting studies (e.g., randomized controlled trials, sample size, duration) and outcome robustness (e.g., magnitude and consistency of weight loss, metabolic improvements, safety profile). Interventions with unpublished or preliminary data but consistent RCT-level results were also graded as high quality if internal validity was strong. Abbreviations: CV, cardiovascular; D1:H11, denotes resolution of ≥ 1 component of metabolic dysfunction in addition to histological improvement in MASH; MASH, metabolic dysfunction-associated steatohepatitis; RCT, randomized controlled trial; T2D, type 2 diabetes

**Table 6:** Precision pharmacotherapy in obesity: evidence-based selection by complication phenotype

Condition (detailed)	Preferred pharmacotherapy (dosage/route)	Efficacy on disease outcome	Evidence source
ASCVD (post-MI, stroke, PAD)	Semaglutide2.4 mg	↓ MACE − 20% (CV death, nonfatal Miocardial Infarction (MI), stroke) by 20% (HR 0.80; 95% CI 0.72–0.90) and all-cause mortality by 19% in 17,604 people with obesity and established ASCVD but without diabetes	[4]
HFpEF (LVEF > 50%, NYHA II-III, obesity-related)	Semaglutide2.4 mg	KCCQ score + 7.8 pts; 6-minute walk distance (6MWD) (+ 20.3 m), reduced C Reactive Protein (CRP) and body weight (− 13.3% vs. − 2.6%)	[5]
	Tirzepatide 15mg	KCCQ score + 6.9 pts (placebo subtracted); 6MWD (18.3 m; 95% CI, 9.9–26.7); tirzepatide significantly lowered the composite of CV death or HF hospitalization (HR 0.62; 95% CI 0.41–0.95), driven by reduced HF hospitalizations (HR 0.54; 95% CI 0.34–0.85), with no significant effect on CV death.	[11]
MASLD/MASH (biopsy-proven or imaging-based)	Semaglutide2.4 mg	Phase III ESSENCE trial: At 72 weeks, semaglutide 2.4 mg led to MASH resolution without fibrosis worsening in 62.9% vs. 34.3% (placebo), fibrosis improvement without MASH worsening in 36.8% vs. 22.4%, and achievement of both endpoints in 32.7% vs. 16.1% (*P* < 0.001 for all).	[55]
	Tirzepatide 15mg	Phase II SynERGY-NASH, at 52 weeks, tirzepatide 5–15 mg led to MASH resolution in 44–62% of patients vs. 10% with placebo (*P* < 0.001). Fibrosis improved in 51–55% vs. 30% with placebo. Gastrointestinal side effects were the most common and mostly mild or moderate.	[56]
T2D (HbA1c ≥ 6.5%)	Tirzepatide15 mg; Semaglutide 2.4 mg; Liraglutide 3 mg	Tirzepatide: ↓ HbA1c −2.8%; Semaglutide↓ HbA1c %−1.6% liraglutide: ↓ HbA1c −1-1.2%	[29, 31]
Prediabetes (FPG 100–125 mg/dL, HbA1c 5.7–6.4%)	Tirzepatide; Semaglutide 2.4 mg; Liraglutide 3.0 mg	tirzepatide: 93.3% reverted to normoglycemia; semaglutide: 81% reverted to normoglycemia; liraglutide 69.2% reverted to normoglycemia	[2, 22, 34]
CKD Stage 2–3 (eGFR 30–89)	Semaglutide 2.4 mg	In SELECT, semaglutide 2.4 mg reduced a PRESPECIFIED composite renal endpoint by 22% vs. placebo, alongside lower CV events and mortality, supporting its use in obesity-related CKD even without diabetes. Reported safe in dialysis patients with obesity (observational study), currently not permitted in clinical practice	[17, 18]
	Tirzepatide 15 mg	Although not yet tested in renal outcome trials, tirzepatide improved eGFR and UACR in SUMMIT (HFpEF + obesity), with supportive biomarker changes in SURMOUNT-1, suggesting potential renal benefit pending further evidence.	[10, 11]

This table summarizes the preferred anti-obesity pharmacologic agents for major obesity-related complications, based on randomized controlled trials. For each condition, key outcomes (e.g., reduction in cardiovascular events, liver fibrosis improvement, glycemic normalization, renal protection) are reported along with the studied dose and trial reference. Arrows indicate direction of effect (↑/↓), and effect sizes are shown where available. Evidence quality is based on study design and prespecified endpoints, with priority given to placebo-controlled phase 3 RCTs Abbreviations: ASCVD = atherosclerotic cardiovascular disease; CKD = chronic kidney disease; CRP = C-reactive protein; CV = cardiovascular; eGFR = estimated glomerular filtration rate; FPG = fasting plasma glucose; HbA1c = hemoglobin A1c; HFpEF = heart failure with preserved ejection fraction; HR = hazard ratio; KCCQ = Kansas City Cardiomyopathy Questionnaire; LVEF = left ventricular ejection fraction; MACE = major adverse cardiovascular events; MASLD = metabolic dysfunction-associated steatotic liver disease; MASH = metabolic dysfunction-associated steatohepatitis; MI = myocardial infarction; NYHA = New York Heart Association; RCT = randomized controlled trial; T2D = type 2 diabetes; UACR = urine albumin-to-creatinine ratio

GLP-1 RAs and dual GIP/GLP-1 agonists exert favorable effects on inflammation, endothelial function, and lipoprotein metabolism, some of which extend beyond weight loss alone [73]. Therefore, therapeutic choices should reflect both anticipated weight reduction and compound-specific benefits.

Weight loss targets must be individualized based on disease burden, age, phenotype, and treatment preferences. Below, we categorize available interventions by average weight loss achieved.

### Weight loss milestone: 5–10%

#### Liraglutide 3.0 mg

~8% weight loss in SCALE Obesity and Prediabetes [12, 74]. **It should be preferred** in early disease or when once-daily administration is acceptable.

#### Naltrexone/Bupropion

Pooled data from COR trials show ~ 5% loss and reduced cravings [27, 75]. It **should be preferred** in emotional eating phenotypes with low cardiometabolic risk.

#### Phentermine/Topiramate ER (low-mid doses 7.5 mg + 46.0 mg)

CONQUER showed − 7.8% weight loss [60]. It **may be considered** when CV risks are controlled.

#### Orlistat (120 mg)

XENDOS reported ~ 2.9 kg placebo-adjusted loss over 4 years [14]. It **may be considered** when systemic agents are contraindicated.

### Weight loss milestone: 10–20%

#### Tirzepatide 5–10 mg

SURMOUNT-1 reported 16% loss [2]. It **should be preferred** when the higher weight loss is needed, OSA, T2D, and HF.

#### Semaglutide 2.4 mg

STEP 1 showed 14.9% loss over 68 weeks [3]. It **should be preferred** for ASCVD, HF, OA, T2D and MASLD/MASH.

#### Phentermine/Topiramate ER (full dose 15.0 mg + 92.0 mg)

Up to 10.9% loss at 56 weeks in EQUIP [16]. It **may be considered** in patients needing rapid, potent weight loss.

### Weight loss milestone: >20%

#### Tirzepatide 15 mg

Achieved 20.9% loss in SURMOUNT-1 [2]. In SURMOUNT-5, tirzepatide outperformed semaglutide 2.4 mg with − 20.2% vs. − 13.7% body weight reduction and superior responder rates [2, 76]. It **should be preferred** when the weight burden is high, OSA, T2D, and HF.

**Semaglutide 7.**2 mg: **It was not available on the market at the time of publication.** In the STEP-UP trial, semaglutide 7.2 mg achieved 20.7% weight loss in adults with obesity, with one in three participants losing ≥ 25% of body Weight. While tolerability was slightly reduced, safety remained acceptable, supporting dose escalation as a viable intensification strategy beyond 2.4 mg [77]. It **may be considered** for intensification when approved.

**CagriSema 2.4/2.4 mg: It was not available on the market at the time of publication.** In the REDEFINE 1 trial, coadministration of cagrilintide 2.4 mg (a long-acting amylin analog) and semaglutide 2.4 mg (CagriSema) resulted in a 20.4% mean Weight loss at 68 weeks in adults with overweight or obesity. Over half of the participants lost ≥ 20% weight, and nearly one in five lost ≥ 30% of their body weight. While gastrointestinal adverse events were frequent (79.6%), they were mainly transient and mild to moderate, supporting CagriSema as a highly effective future option for weight management beyond current monotherapies [78]. It **may be considered** for intensification when approved.

This therapeutic milestone’s system enables clinicians to match treatment intensity to disease severity and patient goals. Tirzepatide and semaglutide are appropriate for those targeting ≥ 10% loss, while surgery remains the most effective option. Selection should account for efficacy, tolerability, cost, accessibility, and contraindications.

### If additional weight loss and reduction of weight-Related complications are needed

Bariatric surgery represents the most effective intervention for sustained weight loss and metabolic improvement in people with severe obesity and has demonstrated consistent benefits across a wide range of obesity-related complications. Long-term data confirm robust effects on mortality, cardiometabolic outcomes, renal disease, T2D progression, musculoskeletal function, sleep apnea, liver disease, and cancer risk. (See Supplementary Material for Clinical Takeaways).

**Cardiovascular Disease and Heart Failure**

In the Swedish Obese Subjects (SOS) study, bariatric surgery reduced all-cause mortality by 29% 0.71 (95% CI, 0.54–0.92), and cardiovascular mortality by 53% 0.47 (95% CI, 0.29–0.76), over a median of 14.7 years compared to matched controls [79]. A meta-analysis of 174,772 participants showed a 49.2% reduction in hazard rate of death (95% CI 46–51, *p* < 0001), with median life expectancy increased by 6.1 years (95% CI 2–6) compared to usual care. The treatment effect was substantially larger for people with diabetes, indicated by an I² of 95.7% and *p* < 0001 [80]. It **may be considered in** T2D or high CV risk with BMI ≥ 35.

**Chronic Kidney Disease (CKD)**

Surgical weight loss significantly reduces albuminuria and the progression to end-stage renal disease. The SOS study reported reduced end-stage renal disease (ESRD) incidence after surgery (adjusted hazard ratio (HR) = 0.27; 95% CI 0.12–0.60; *p* = 0.001) [81]. A meta-analysis confirmed improvements in eGFR and proteinuria even among non-diabetic patients [82]. Although this improvement declined somewhat over the 5-year follow-up, eGFR stayed above baseline levels for the CKD group. Notably, eGFR also improved in patients with CKD not directly caused by obesity, supporting the use of surgery in obesity-related CKD when conservative treatments are insufficient. It **may be considered** when Obesity and CKD progress despite medical therapy.

**Type 2 Diabetes (T2D) and Prediabetes**

Bariatric surgery can induce long-term T2D remission. In randomized trials, up to 50% of patients maintained remission 10 years after biliopancreatic diversion, while remission after Roux-en-Y gastric bypass was less durable [83]. Microvascular and macrovascular outcomes also improve significantly [72]. Guidelines recommend surgery for patients with BMI ≥ 35 regardless of glycemic control and for BMI 30–34.9 when medical therapy is insufficient [84].

In prediabetes, surgery halts disease progression. In a cohort of 175 people, 82% achieved remission at 1 year, with 58% maintaining remission at 4 years. Predictors included sleeve gastrectomy, younger age, and male sex [85]. It **should be preferred** for T2D with BMI ≥ 35 or ≥ 30 ² if uncontrolled.

**Type 1 Diabetes (T1D)**

Though remission is not expected, bariatric surgery reduces insulin requirements, weight, and HbA1c in T1D patients. In this 5-year retrospective analysis of patients with T1D undergoing either sleeve gastrectomy (SG) or Roux-en-Y gastric bypass (RYGB), total Weight loss reached 14.2% after SG and 22.6% after RYGB. Importantly, median daily insulin requirements decreased substantially from 140.5 units preoperatively to 77.5 units at 1 year, and remained lower at 90.0 units at 5 years. The reduction was more pronounced and durable following RYGB compared to SG [86]. Careful perioperative management is essential due to the risk of diabetic ketoacidosis. It **may be considered** in obesity with T1D to improve obesity and its related complications and reduce insulin requirements.

**Metabolic Dysfunction–Associated Steatotic Liver Disease (MASLD/MASH)**

A comprehensive systematic review and meta-analysis assessed the histological effects of bariatric surgery on nonalcoholic fatty liver disease (NAFLD) in patients, analyzing 3,093 paired liver biopsies from 32 cohort studies. Bariatric surgery was associated with significant improvements in hepatic histology: steatosis resolved in 66% of cases, lobular inflammation in 50%, hepatocellular Ballooning in 76%, and fibrosis in 40%. The overall NAFLD activity score notably decreased (mean difference − 2.39; 95% CI, − 3.20 to − 1.58; *P* < 0.001), supporting the capacity of surgery-induced Weight loss to reverse key pathological features of NAFLD. However, 12% of patients developed new or worsening liver disease, particularly fibrosis [87]. While no RCTs directly comparing surgery and pharmacotherapy for NASH exist, a recent network meta-analysis incorporating 24 surgical and non-surgical trials confirms the superior efficacy of bariatric surgery in achieving NASH resolution (79% vs. 29% with drugs), with comparable effects on fibrosis improvement. These findings highlight the need to consider surgery among therapeutic options for eligible patients with NASH [88]. **It should be preferred** in advanced MASH, especially with fibrosis.

**Osteoarthritis (OA)**

Bariatric surgery improves OA symptoms, reduces pain, and enhances function. A prospective study of 82 patients observed significant improvement at 6 and 12 months post-RYGB [89]. In the LABS-2 study, up to 77% of people reported clinically essential improvements in knee or hip pain at 3 years, with most benefits persisting at 7 years (65–72%). Although some decline was observed over time, meaningful gains in mobility and function remained substantial, supporting bariatric surgery as an effective strategy for managing obesity-related osteoarthritis [90]. It **should be preferred** in disabling OA where weight loss is a goal.

**Obstructive Sleep Apnea (OSA)**

Bariatric surgery has a significant impact on the severity of OSA in people with obesity, as demonstrated by a 2023 meta-analysis of 32 studies including 2,310 patients. The procedure was associated with substantial reductions in body mass index (–11.9 kg/m²), apnea-hypopnea index (–19.3 events/hour), and respiratory disturbance index (–33.9 events/hour), with approximately 65% of patients achieving remission of OSA. These findings confirm the efficacy of bariatric surgery in alleviating OSA symptoms; however, the incomplete remission rate underscores that obesity is not the sole driver of OSA, and anatomical or neuromuscular factors likely contribute to its persistence [91]. It **should be preferred** in moderate-severe OSA with obesity.

**Cancer Risk Reduction**

Surgical Weight loss reduces the incidence of obesity-related cancers. A Swedish matched cohort study found a 33% reduction in cancer risk over 10 years [92]. The SPLENDID trial demonstrated a 32% lower incidence of obesity-related cancers and a 48% reduction in cancer-related mortality after RYGB or sleeve gastrectomy [93]. There is no evidence of increased cancer risk post-surgery, but long-term nutritional monitoring is essential. It **may be preferred** in younger people with high lifetime cancer risk.

## Weight loss for people living with obesity without clinically manifest complications (OWCMC)

Addressing obesity early, even before the onset of clinically manifest complications, has gained traction in recent years, bolstered by epidemiological trends and mechanistic insights into adipose tissue dysfunction. The 2024 Lancet Commission on the diagnosis and management of obesity formally introduced the notion of a “preclinical obesity” stage, analogous to other chronic diseases such as prediabetes or prehypertension. Within this framework, people with excess adiposity but without evidence of reduced organ/tissue function due to obesity or significant, age-adjusted limitations of day-to-day activities would still warrant intervention, primarily through structured lifestyle modification rather than pharmacotherapy or surgery, to avert the progression toward metabolic dysfunction and end-organ damage [94]. This position aligns with the broader shift toward preventive medicine and early risk stratification, but it has drawn significant criticism. The European Association for the Study of Obesity (EASO) has expressed strong opposition to the preclinical obesity construct, cautioning against the risk of further stigmatization, overmedicalization, and potential overtreatment of otherwise healthy people. EASO emphasizes the need for careful phenotyping and patient-centered risk-benefit evaluation, suggesting that a BMI-centric approach without clinical context risks doing more harm than good [95]. Further complicating the debate are recent data from Klein et al., published in *Cell Metabolism* in 2024, which challenge the assumption that a truly “healthy” obesity phenotype exists. In a comprehensive multi-organ metabolic profiling study of people rigorously categorized as Metabolically Healthy Obese (MHO) versus Metabolically Unhealthy Obese (MUO), the authors found shared pathophysiological alterations across groups, including insulin resistance in skeletal muscle, subclinical inflammation, altered lipid turnover, oxidative stress, and cardiac structural changes. While MHO people were free from overt disease by standard diagnostic thresholds, they nonetheless exhibited quantifiable biological disturbances that suggest a continuum of risk rather than a dichotomy. These findings undermine the binary classification of MHO versus MUO and highlight the progressive, system-wide impact of adiposity, even without diagnosed disease [96]. Importantly, Marcus et al. have further highlighted that almost all organ systems are affected by obesity from an early age. Inflammatory markers, immune dysfunction, and increased risk for T2D, cardiovascular disease, autoimmune conditions, and even early mortality are observed in children with obesity, often before any clinical signs of disease emerge. These effects persist throughout life unless weight normalization occurs before puberty [97]. From a therapeutic standpoint, early interventions, including pharmacologic ones, may offer benefits beyond metabolic disease prevention, improving aspects such as physical function, body image, psychological well-being, and overall health-related quality of life (HRQoL). Both the STEP 1 and SURMOUNT-1 trials, evaluating semaglutide and tirzepatide, respectively, demonstrated clinically meaningful improvements in HRQoL measures as assessed by standardized tools (e.g., IWQOL-Lite, SF-36), even in people without diabetes or established cardiovascular disease [2, 3]. These results support a broader definition of treatment success in obesity, including patient-centered outcomes and recognizing quality of life as a legitimate therapeutic target. Thus, while the terminology and conceptual boundaries of “preclinical obesity” may remain contentious, the authors of this paper advocate for proactive management of obesity, regardless of whether classical complications are present or absent. Disease-modifying interventions, including lifestyle counselling, behavioral therapy, and in selected cases, AOMs, may interrupt the trajectory towards overt disease, minimize cumulative metabolic burden, and enhance well-being. Importantly, treatment decisions should be individualized, respectful of patient values and preferences, and based on comprehensive phenotyping rather than BMI thresholds alone.

## Stratification in vulnerable ages

### Pediatric obesity: efficacy, safety, and approvals

Childhood obesity is strongly associated with earlier onset of cardiometabolic deterioration, mechanical complications (OA, and OSA), and psychosocial burden. Management should focus on behavioral and family-based strategies. Effective interventions include limiting sugar-sweetened beverages, promoting active play, and ensuring healthy foods and age-appropriate portions at home. Active caregiver involvement is essential, with family-centered behavioral counselling shown to be the most effective approach. Both the American Academy of Pediatrics (AAP) and the European Childhood Obesity Group emphasize that lifestyle modification remains the cornerstone of treatment in this age group) [98]. Besides, even the EASO-EFAD statement highlights the role of lifestyle intervention for the treatment of obesity in children [99]. While lifestyle modification remains first-line, its limited long-term success in severe obesity has led to expanded pharmacological options [100]. The 2023 AAP guideline recommends that children aged between 8 and 11 age with obesity may be offered weight loss pharmacotherapy, according to medication indications, risks, and benefits, as an adjunct to health behavior and lifestyle treatment [101]. Similarly, the 2025 Canadian Clinical Practice Guidelines (CPCG) recommend pharmacological intervention in combination with behavioral and psychological interventions [100]. Both guidelines recommend prescription of GLP1RA (liraglutide and semaglutide); and consider the off-label prescription of metformin even though findings on the Weight loss effect are inconsistent. While the 2023 AAP CPG recognize orlistat is not commonly prescribed in clinical practice owing to the Limited tolerability, the CPCG suggests against the use of the molecule. The 2023 AAP CPG also recommends phentermine/topiramate. As of 2025, five agents, semaglutide, liraglutide, orlistat, setmelanotide and metreleptin are FDA and EMA-approved for chronic weight management in adolescents ≥ 12 years, while phentermine/topiramate is exclusively FDA approved. Setmelanotide is FDA and EMA approved in children > 2 years old in selected cases of genetic and syndromic obesities. In July the FDA extended liraglutide’s approval to include children aged 6 to 11years old. Prescribing typically requires pediatric endocrinology authorization. Nonetheless, barriers remain, including access, reimbursement, and provider experience disparities. Multidisciplinary care remains critical for growth monitoring, psychosocial assessment, and treatment adherence. While pharmacotherapy may delay surgery, it does not replace its role in adolescents with severe obesity and comorbidities. Surgery remains indicated for youth with BMI ≥ 120% of the 95th percentile or BMI ≥ 40 kg/m², or ≥ 35 kg/m² with severe complications [100]. Pharmacological therapy has transformed pediatric obesity management. Semaglutide currently shows the highest efficacy, followed by liraglutide and phentermine/topiramate [102]. Used within multidisciplinary care models, these agents yield significant improvements in BMI, metabolic health, and quality of life. The big issues are about bone health and neurodevelopment in children and adolescents, and about pubertal development in children. Ongoing long-term studies in children and adolescents, i.e. the STEP TEENS Weight Maintenance NN9536-7752 (clinicaltrials.gov), will ascertain the effect on bone growth and maturation. Ongoing success will rely on early identification, individualized therapy, and long-term support (Figs. 1 and 2; Table 7) (see Supplementary Material for Clinical Takeaways).

**Table 7:** Anti-obesity medications approved for pediatric use: indications, efficacy, and clinical considerations

Agent	Age approved	Mean BMI reduction	Key adverse events	Clinical role	Evidence source
Semaglutide (2.4 mg OW)	≥ 12 years (US/EU)	−16.1%	GI (nausea, vomiting), gallstones	First-line AOM for adolescents with severe obesity (also improvement in HbA1c, ALT and WC)	[113]
Liraglutide (3.0 mg daily)	(6 to < 12 years) (EU); ≥12 years (US)	−5.8%; −4.64%	GI (nausea, vomiting)	Alternative to semaglutide especially for the (6 to < 12 years) patients	[109, 112]
Phentermine/Topiramate ER	≥ 12 years (US)	10% (high dose)	Mood changes, potential for depression	Oral option for hyperphagic youth, improvement in triglycerides and HDL.	[115]
Orlistat	≥ 12 years (US)	~ 3–5%	GI effects (steatorrhea, oily stools)	Rarely used; consider if systemic agents are contraindicated (not approved in Canada)	[114]
Setmelanotide	≥ 2 years (monogenic only)	Variable (only in monogenic forms)	Hyperpigmentation, nausea, injection-site reactions	Specialist use only for confirmed genetic obesity	[107]

This table summarizes the anti-obesity medications (AOMs) currently approved for pediatric patients with obesity in the US and/or EU, including age indications, expected treatment efficacy based on BMI reduction or weight loss (kg or SDS), common adverse effects, and their proposed clinical role. Semaglutide and liraglutide are glucagon-like peptide-1 receptor agonists (GLP-1 RAs), increasingly used in adolescents with severe obesity, while phentermine/topiramate extended release (ER) and orlistat represent older oral options. Setmelanotide, a melanocortin-4 receptor agonist, is reserved for rare cases of genetically confirmed monogenic obesity. The table incorporates pivotal evidence sources for each medication, and highlights tolerability concerns such as gastrointestinal side effects, mood changes, or injection-site reactions. Clinical role designations are based on regulatory indications, trial outcomes, and expert consensus.Abbreviations: AOM, anti-obesity medication; BMI, body mass index; GI, gastrointestinal; SDS, standard deviation score; ER, extended release. ALT, alanine aminotransferase; WC, Waist Circumference; HDL, High Density Lipoprotein

### 2–6 years

#### Pharmacotherapy

While most pediatric obesity cases are polygenic and environmentally mediated, onset of severe obesity in infancy, particularly when accompanied by hyperphagia, poor satiety, may be a marker of monogenic or syndromic forms of features. Recognizing these rare cases is essential, as targeted therapies (e.g., setmelanotide) may be effective in specific genotypes. Setmelanotide, a selective agonist of the MC4R, is approved by the FDA and EMA for the treatment of obesity and control of hunger in Bardet-Biedl Syndrome or biallelic pro-opiomelanocortin (POMC), proprotein convertase subtilisin/kexin type 1 (PCSK1), or leptin receptor (LEPR) deficiency in affected children above age 2 years-old [103]. *The VENTURE Phase III trial* studied children aged 2 to under 5 with POMC, LEPR defects or BBS, treated for 1 year [104]. The study found that about 83% achieved a ≥ 0.2 reduction in BMI z‑score and mean BMI reductions of the 26% for patients with POMC/LEPR deficiencies and of the 10% for BBS. The hunger score ameliorated significantly in about ~ 91% of the treated patients. Most of the patients experience skin hyperpigmentation that is the main cause of treatment discontinuation.

The early initiation of Metreleptin, a recombinant analog of human leptin, in generalized lipodystrophy patients is essential to delaying or preventing metabolic complications, lowering HbA1c, triglycerides, proteinuria, and improving liver health [105]. This drug is approved from age two.

Obesity that begins before the age of 2, characterized by a rapid increase in BMI (> 2 standard deviations above the mean) during the first year of life followed by either a plateau or a continued steady rise, should prompt further medical evaluation for **monogenic obesity** [103]. Referral for genetic testing is advised in the presence of: (1) Severe obesity before age 5 (BMI > 97th percentile), especially from infancy; (2) Hyperphagia; (3) Family history of early-onset severe obesity in multiple first-degree relatives [106]; (4) Associated features: dysmorphisms, developmental delay; (5) Dysmorphic features, developmental delay, or endocrinopathies (e.g., central hypothyroidism, GH or ACTH deficiency) [107] and (6) Normal or accelerated linear growth (suggesting genetic rather than hypothalamic etiology, especially in MC4R mutated-patients); however, if a GH deficit is present, the phenotype is lower height.

The diagnostic work-up should begin with thorough clinical phenotyping, assessing growth patterns, feeding behavior, and neurodevelopment. Endocrine evaluation is essential to exclude hypothalamic or syndromic causes. In parallel, next-generation sequencing panels targeting *MC4R*, *LEPR*, *POMC*, *PCSK1*, and *SIM1* should be performed in coordination with a specialized pediatric endocrinology unit.

### 6–12 years

Pharmacotherapy in this age range should only be considered in specialized centers, with intensive lifestyle therapy. Long-term safety data are limited.

#### Setmelanotide

Approved from age 2, but limited to specific genetic disorders (e.g., biallelic POMC, LEPR, Bardet-Biedl syndrome) [108], and may be used in this age range (genotype-restricted).

The EMANATE (NCT05093634) trial is a multicenter, phase 3, randomized, double-blind, placebo-controlled study designed to evaluate the efficacy and safety of setmelanotide in individuals aged 6 to 65 years with obesity due to rare heterozygous variants in genes involved in the melanocortin-4 receptor (MC4R) pathway. The study includes several parallel sub-studies targeting distinct genetic subpopulations, including POMC, PCSK1, LEPR, SRC1, SH2B1, and PCSK1 N221D variants. Approximately 400 participants were randomized to receive either setmelanotide or placebo over 52 weeks. The primary endpoint is the proportion of participants achieving a clinically meaningful response, defined by weight loss and/or reduction in hyperphagia. EMANATE aims to extend the indications of setmelanotide beyond biallelic POMC, PCSK1, and LEPR deficiencies, potentially addressing a broader spectrum of genetically driven obesity. Findings are expected by mid 2026.

**Liraglutide 3.0** mg was recently approved in the EU for use in people 6 years old. In a phase 3 trial, children aged 6 to < 12 years treated with liraglutide experienced a − 7.4% point difference in BMI change vs. placebo over 56 weeks (− 5.8% vs. + 1.6%, *P* < 0.001). Nearly half achieved ≥ 5% BMI reduction [109]. Gastrointestinal side effects were frequent but manageable.

The **Semaglutide Pediatric Trial (NCT05064735)** is a Phase 3b study currently ongoing involving approximately 400 participants aged 6–17. It is a randomized, double-blind, placebo-controlled study with a duration of 104 weeks. All participants receive semaglutide for ≥ 3 years, and the study evaluates long-term safety and effectiveness.

#### Orlistat

Not approved in this range of age [100].

### 12–18 years

Adolescents with severe obesity (≥ 120% of the 95th percentile) and comorbidities such as prediabetes, PCOS, or MAFLD may benefit from pharmacotherapy and for them the pharmacological treatment in particular with Phentermine/Topiramate ER is cost effective [110, 111]. GLP-1 RAs are particularly suited for those with increased hunger, insulin resistance, or MASH. Multidisciplinary support, including nutrition, behavioral therapy, and psychological screening, is critical due to the elevated risk of eating and mood disorders during adolescence.

#### Setmelanotide

may be used in this age range (genotype-restricted) [108].

#### Liraglutide 3.0 mg

Approved by FDA/EMA from age 6. In a key RCT, there were notable reductions in BMI SDS and higher percentages of participants achieving ≥ 5% and ≥ 10% Weight loss compared to placebo and the BMI standard-deviation score at week 56 (estimated difference, − 0.22; 95% confidence interval [CI], − 0.37 to − 0.08; *P* = 0.002) [112], but modest reduction in main CVD factors over 56 weeks of treatment.

#### Semaglutide 2.4 mg

Approved by FDA/EMA from age 12. In the STEP TEENS trial, adolescents achieved a − 16.1% mean BMI reduction vs. + 0.6% in the placebo group. 73% achieved ≥ 5% weight loss and with significant amelioration of CVD factors (e.g. waist circumference, ALT and HbA1c) and no adverse impact on pubertal development and bone health [113].

#### Tirzepatide

The SURMOUNT-ADOLESCENTS (NCT06075667) and (NCT06439277) are Phase 3 studies recruiting ~ 150 and 300 adolescents who will be treated with tirzepatide for 90 and 76 weeks, respectively.

#### Orlistat

FDA approved from age 12; infrequently used due to GI adverse events [114].

#### Phentermine/Topiramate ER

FDA-approved for ≥ 12 years in the U.S.; not approved by EMA. In adolescents with obesity (mean age 14.0 years, BMI 37.8 kg/m²), phentermine/topiramate significantly reduced BMI at 56 weeks versus placebo by − 10.4 and − 8.1% points for the top and mid doses, respectively (*P* < 0.001). Improvements in triglycerides and HDL-C also favored active treatment. Adverse events were common but comparable to placebo; serious events occurred in two participants receiving the top dose [115].

**Metformin** is used **off-label** in adolescents with **insulin resistance or PCOS**, with modest weight effects. It may be considered when hyperinsulinemia is a dominant feature but is not a weight-loss medication per se [116].

### Obesity in adults and older people

Obesity manifests differently across age groups. In adults aged 18–65, it is often driven by cardiometabolic or behavioral factors, supporting phenotype-guided pharmacotherapy. In older adults (≥ 65), obesity frequently coexists with sarcopenia, frailty, or chronic pain, warranting treatments that focus on preserving mobility, independence, and quality of life. Therapeutic strategies must be individualized: younger adults may benefit from targeting ASCVD risk, diabetes prevention, or NAFLD; older adults require agents with high tolerability, minimal appetite suppression, and caution to avoid muscle loss and drug interactions (Figs. 1 and 2) (See Supplementary Material for Clinical Takeaways).

### 18–65 years (Adults)

This age group is addressed throughout the manuscript, with treatment considerations integrated across all phenotypes and obesity-related complications.

### > 65 years (Older Adults)

In people ≥ 65 years, the primary aim of pharmacotherapy is to preserve function, reduce obesity-related complications (e.g., osteoarthritis, cardiometabolic risk), and avoid harm. Weight loss is secondary to maintaining muscle mass, nutritional status, and autonomy. Data from RCTs specific to weight outcomes in this population are limited; most safety and efficacy data derive from cardiovascular outcome trials (CVOTs) using AOMs.

**Preferred Agents**:

#### Liraglutide

Proven safe and effective in older adults with diabetes and obesity, with robust CVOT support [34].

**Agents to Avoid**:

#### Naltrexone/Bupropion

May exacerbate agitation, insomnia, and elevate blood pressure.

#### Phentermine and other stimulants

Not recommended in people > 75 due to neuropsychiatric and cardiovascular risks.

#### Orlistat

May cause gastrointestinal side effects and dehydration, which should be avoided in older adults.

### Sarcopenic obesity

Sarcopenic obesity is a distinct phenotype defined by the coexistence of excess adiposity and reduced skeletal muscle mass and/or strength. It is particularly prevalent among older adults and people with chronic diseases such as T2D, CKD, and cardiovascular disease, and is associated with increased risks of frailty, falls, disability, hospitalization, and mortality [117]. The diagnostic challenge lies in the fact that fat mass can obscure the progressive loss of muscle, leading to under recognition when using BMI alone.

The condition arises from the interplay between anabolic resistance, chronic low-grade inflammation, insulin resistance, physical inactivity, and mitochondrial dysfunction. These mechanisms lead to muscle catabolism despite caloric excess, creating a therapeutic paradox: while weight loss may improve metabolic risk, it can further deplete lean mass and compromise physical function if not carefully managed.

In this context, the primary therapeutic goal is not to achieve maximal Weight reduction, but rather to preserve or recover muscle mass and strength. Gradual Weight loss of 5–10% may be beneficial, provided it is accompanied by gains in mobility, improved cardiometabolic parameters, or reductions in visceral adiposity. Rapid or unsupervised weight loss should be avoided, particularly in frail people, due to the risk of disproportionate muscle loss.

Core interventions include a protein intake of ≥ 1.2 g/kg body Weight per day, up to 1.5 g/kg/day during caloric restriction, distributed evenly across meals to optimize anabolic signaling [118]. Supervised, progressive resistance training 2–3 times per week is critical for preserving type II muscle fibers, enhancing strength and balance, and remains the most evidence-based non-pharmacologic intervention in this setting. Micronutrient optimization, especially with ≥ 800 IU/day of vitamin D and adequate calcium, is important for muscle function and fall prevention [119]. The SARC-F questionnaire is a validated tool for routine screening; a score ≥ 4 indicates probable sarcopenia and warrants further assessment with DXA, BIA, handgrip strength, or gait speed testing [120].

Pharmacologic management must support functional preservation and minimize unintended loss of lean mass. Among approved agents, liraglutide 3.0 mg daily has demonstrated modest weight reduction (~ 6%) while maintaining lean mass when paired with adequate protein intake and physical activity [74]. Its cardiovascular safety and slow kinetics make it especially suitable for older or frail people. Orlistat is another suitable option, producing ~ 3–4% weight loss without central appetite suppression. It is particularly appropriate in polymedicated patients, though requires supplementation with fat-soluble vitamins.

More potent agents such as tirzepatide and high-dose semaglutide induce substantial weight loss (~ 15–24%) and reduce both fat and lean mass. However, recent data show that roughly two-thirds of weight loss is derived from adipose tissue, indicating a relatively favorable fat-to-lean mass loss ratio [121]. Nevertheless, their use in sarcopenic obesity requires concurrent resistance training and nutritional support to mitigate functional decline [122].

Caution should be exercised with naltrexone/bupropion, since frail or cognitively impaired people can poorly tolerate neuropsychiatric side effects like agitation, insomnia, and anxiety. In summary, the management of sarcopenic obesity must balance metabolic improvement with the preservation of functional reserve. Lifestyle interventions remain central, and pharmacotherapy should be selected and monitored with attention to lean mass preservation, micronutrient adequacy, and physical performance (Figs. 1 and 2) (see Supplementary Material for Clinical Takeaways).

## Eating behavior phenotypes

### Emotional eating

Emotional eating refers to consuming food in response to negative emotions (e.g., anxiety, sadness, stress) rather than physiological hunger. This behavior is common in people with obesity and contributes to poor weight loss outcomes, high relapse rates, and risk of binge eating disorder. Neurobiologically, it involves hyperactivity in cortico-limbic circuits, such as the amygdala, insula, and orbitofrontal cortex, which can override satiety signals and drive compulsive intake [123, 124]. For this population, pharmacologic success may depend on weight loss and the drug’s effects on reward processing, craving, and disinhibition (Figs. 1 and 2) (see Supplementary Material for Clinical Takeaways).

#### Semaglutide

Semaglutide reduces emotional eating and food cue reactivity and increases cognitive restraint through modulation of hypothalamic and mesolimbic reward circuits [125]. Benefits on quality of life and disordered eating have been reported, though severe emotional dysregulation may require adjunctive therapy. Semaglutide **may be considered** in people with emotional eating, especially when weight loss and improved emotional regulation are desired outcomes.

#### Tirzepatide

Tirzepatide does not directly affect emotional eating but led to improved quality of life scores in SURMOUNT-3; for example, after 72 weeks, adults achieving ≥ 25% weight reduction with tirzepatide saw improvements in the SF-36v2 Role-Emotional score, Mental Health, Physical Functioning, and IWQOL-Lite-CT Psychosocial Composite [126]. Tirzepatide **may be considered** in people with obesity and emotional eating when significant weight reductions are required, although it does not directly modulate reward circuits.

#### Liraglutide

Adding liraglutide to intensive behavioral therapy led to greater short-term improvements in eating behaviors, particularly reductions in dietary disinhibition, shape concern, and global eating disorder psychopathology, compared to intensive behavioral therapy alone, with benefits attenuating by week 52 [127]. Liraglutide **may be considered** as adjunct to structured behavioral interventions in emotional eating, particularly in early treatment phases.

#### Naltrexone/Bupropion

This combo targets reward-driven eating through dual action on hypothalamic POMC and opioid pathways. In the COR-II Phase 3 trial, naltrexone/bupropion significantly improved control of eating compared to placebo, as measured by the Control of Eating Questionnaire. Participants reported fewer and less intense food cravings, and greater ability to resist urges to eat, indicating enhanced regulation of eating behavior [28]. Due to psychiatric risks, careful patient selection is essential. Naltrexone/bupropion **should be preferred** in people with obesity and emotional eating, particularly when dysregulated reward and craving are dominant features.

#### Phentermine/Topiramate ER

This combination has shown efficacy in reducing binge symptoms, often overlapping with emotional eating. Phentermine curbs appetite while topiramate enhances inhibitory control [128]. Cognitive side effects, however, may limit tolerability. Phentermine/topiramate ER **may be considered** with caution in selected people with emotional eating, particularly when binge features are prominent and psychiatric stability is confirmed.

#### Orlistat

Without central action, orlistat does not affect emotional eating. Grilo et al. found that orlistat improved weight loss in patients with obesity and without Binge Eating Disorder (BED) but offered no added benefit in those with BED. Remission of binge eating was achieved mainly through behavioral therapy, not the drug [129]. Orlistat is **not recommended** in people with emotional eating, as it lacks efficacy in modulating relevant behavioral drivers.

### Binge eating disorder (BED)

BED is the most common eating disorder in people with obesity, affecting up to 30% of patients in bariatric and weight management settings [130]. It is characterized by recurrent episodes of uncontrolled eating and significant distress, and is associated with psychiatric comorbidities (e.g., depression, anxiety, and substance use) and increased metabolic risk [131]. Neuroimaging studies reveal dysfunction in corticostriatal-limbic pathways, particularly in the orbitofrontal cortex, nucleus accumbens, and insula [132, 133]. Pharmacologic approaches in BED aim to reduce both weight and binge episodes by targeting reward, satiety, and impulse regulation. At the same time, cognitive behavioral therapy remains first-line, pharmacotherapy is often necessary in moderate-to-severe cases, especially when obesity coexists [134]. (Figures 1 and 2) (See Supplementary Material for Clinical Takeaways).

#### Semaglutide

In a prospective study, Semaglutide decreased binge frequency and craving intensity, and improved Binge Eating Scale scores. These results were consistent in patients with moderate to severe BED, as well as in the entire sample [134]. Although not tested in BED-specific RCTs, it shows promise through modulation of satiety and mesolimbic reward circuits. In participants completing the Control of Eating Questionnaire, Semaglutide resulted in significantly greater weight loss (–14.8% vs. − 2.4%) and better control over cravings, especially for savory, sweet, starchy, and dairy foods, compared to placebo. These improvements appeared as early as week 20 and were associated with Weight reduction through week 104; however, the craving for sweets returned at week 104 [135]. Semaglutide **may be considered** in people with BED and obesity when weight loss and craving control are therapeutic goals. Despite limited BED-specific RCTs, it may be beneficial through satiety and reward modulation.

#### Tirzepatide

Tirzepatide led to improved quality of life scores in SURMOUNT-3 [136]. Preclinical data suggest GIP agonism may reduce stress-induced food seeking [137]. Tirzepatide is being evaluated for treating BED in the planned phase 3 trial LIBERATE (NCT06847399), not yet recruiting. This randomized, double-blind study will compare tirzepatide to placebo and lisdexamfetamine dimesylate in adults with BED, representing the first targeted investigation of tirzepatide in this clinical population. Tirzepatide **may be considered** in people with BED and obesity, particularly when substantial weight loss is prioritized. Dedicated clinical data are pending.

#### Liraglutide

In a 17-week pilot RCT, liraglutide 3.0 mg led to greater weight loss (–5.2% vs. − 0.9%, *p* = 0.005) but no significant difference in binge remission or reduction in binge episodes compared to placebo. Both groups showed improvements in binge eating episodes, highlighting the need for further research in BED [138]. Liraglutide **may be considered** in people with BED primarily for weight loss, but current evidence does not support its use for binge remission.

#### Naltrexone/Bupropion

This combination modulates dopaminergic and opioidergic reward pathways, reducing craving and compulsive eating. Secondary analyses show the benefits of binge frequency and global impressions of severity [139]. It may suit patients with emotion-driven or addictive eating but requires careful use in those with psychiatric or cardiovascular risks.

In a 12-week randomized, double-blind, placebo-controlled trial of 89 adults with BED, naltrexone/bupropion did not significantly reduce binge-eating frequency or remission rates compared to placebo. However, it led to significantly greater Weight loss: 27.9% of treated participants achieved ≥ 5% Weight reduction versus 6.5% with placebo. Obesity status did not influence treatment effects. These findings suggest that while naltrexone/bupropion is ineffective for binge control, it may be beneficial for weight management in BED patients [140]. Further studies should explore combination strategies with psychotherapy and long-term outcomes [140].

Other results indicated that both naltrexone/bupropion and placebo groups experienced significant reductions in binge-eating episodes, eating-disorder psychopathology, and depression during the treatment period. However, these improvements did not differ significantly between the two groups [141]. Naltrexone/bupropion **may be considered** in BED patients where emotional or addictive eating coexists with obesity but should not be relied upon to reduce binge episodes. Combination with psychotherapy should be considered.

#### Orlistat

In a 24-week randomized, double-blind trial, 89 adults with BED and BMI ≥ 30 kg/m² received orlistat 120 mg or placebo three times daily with a mildly hypocaloric diet. Orlistat led to greater weight loss (− 7.4% vs. −2.3%; *p* = 0.0001) and improved eating disorder symptoms (EDI-2 score; *p* = 0.011) compared to placebo. However, due to its peripheral mechanism of action and frequent gastrointestinal side effects, orlistat does not address the core pathophysiology of BED and is poorly tolerated in people with disordered eating. It is **not recommended** as a first-line therapy in BED, although modest weight loss may confer secondary psychological benefit [142].

## Special populations

### Cancer safety

Obesity is a recognized risk factor for multiple malignancies, including endometrial, postmenopausal breast, colorectal, pancreatic, esophageal adenocarcinoma, and hepatocellular carcinoma. Large-scale epidemiologic cohorts and Mendelian randomization studies have established a dose-dependent association between adiposity and cancer incidence and mortality [143]. Mechanistically, this relationship is driven by chronic low-grade inflammation, insulin resistance, increased levels of insulin and IGF-1, elevated estrogen and adipokines such as leptin, and impaired immune surveillance, all of which contribute to tumor initiation, proliferation, and evasion of apoptosis [144].

Intentional Weight loss via lifestyle intervention or bariatric surgery improves many of these pathways and has been associated with reduced cancer risk. Notably, metabolic surgery leads to long-term reductions in systemic inflammation, improvements in insulin sensitivity, normalization of sex hormone levels, and enhanced immune competence. In the SPLENDID cohort, bariatric surgery was associated with a 32% lower incidence of obesity-related cancers and nearly 50% reduction in cancer-specific mortality over a 10-year follow-up [93].

As AOMs are increasingly used in clinical practice, long-term oncologic safety has become important, especially for incretin-based therapies. Initial concerns arose from rodent models suggesting a risk of C-cell hyperplasia and thyroid malignancy, though these findings have not consistently translated to humans. A 2025 meta-analysis by Silverii et al. that pooled 50 randomized trials, GLP-1 RA were associated with a modest but significant increase in thyroid cancer risk (MH-OR 1.55, 95% CI 1.05–2.27), particularly in longer-duration studies. No other cancer types showed significant associations [145]. Current regulatory guidance maintains that GLP-1 RAs and GIP-GLP1RA are **contraindicated** in people with a personal or family history of medullary thyroid carcinoma or multiple endocrine neoplasia type 2 (MEN2).

Pancreatic cancer has also been a historical concern with incretin-based agents. However, Data from 37 RCTs and 19 real-world studies involving over 46,000 patients showed no significant increase in pancreatic cancer risk with GLP-1RAs (OR 0.25, 95% CI 0.03–2.24; *P* = 0.21) compared to placebo [146].

Likewise, no signals have emerged for increased risk of gastrointestinal malignancies. In the SELECT trial involving over 17,000 participants followed for 3.3 years, Semaglutide was not associated with higher incidence of colorectal, gastric, or esophageal cancers [4]. However, a small increase in colorectal cancer was detected in short-term trials (MH-OR 1.27, 95% CI 1.03–1.57), potentially due to increased diagnostic procedures prompted by GLP-1 RA gastrointestinal side effects. GLP-1 RA were not associated with an overall increased cancer risk (MH-OR 1.05, 95% CI 0.98–1.13). A significant reduction in uterine cancer was noted in obesity-focused trials (MH-OR 0.24, 95% CI 0.06–0.94), but not in diabetes trials [145].

Tirzepatide has undergone similar scrutiny. A 2025 meta-analysis of 12 RCTs involving more than 25,000 participants showed no elevated risk of any cancer type, including thyroid (RR 1.07, 0.22–5.12 *p* = 0.93), pancreatic (RR 0.85, 0.10–7.43 *p* = 0.89), or gastrointestinal malignancy (RR 0.73, 0.26–2.04 *p* = 0.54) [147]. While rodent studies previously reported C-cell hyperplasia with tirzepatide, human data to date do not support an increased risk. Long-term follow-up from the SURPASS and SURMOUNT programs is ongoing but has not raised safety concerns to date.

Other pharmacotherapies, such as naltrexone/bupropion, have not been associated with increased cancer risk. The LIGHT trial was terminated early, limiting long-term conclusions, but no oncogenic signals were observed during follow-up [15]. Similarly, no evidence implicates phentermine/topiramate ER in carcinogenesis, though long-term oncologic surveillance data remain sparse. Due to its central nervous system effects and teratogenicity, topiramate should be used cautiously, particularly in populations with neuroendocrine tumor predisposition, despite no direct mechanistic or clinical link [60].

Orlistat, a gastrointestinal lipase inhibitor, has occasionally been associated with case reports of colon and breast cancer. However, a large population-based analysis using UK registry data over 10 years found no increase in colorectal cancer risk [148]. Nevertheless, long-term orlistat use may reduce absorption of fat-soluble vitamins (A, D, E), with potential theoretical implications for immune competence and cancer defense. Patients on extended orlistat therapy should undergo periodic nutritional assessment [149].

In conclusion, the current evidence does not demonstrate a meaningful increase in cancer incidence with approved AOMs. The possible association between GLP-1 RAs and thyroid cancer warrants ongoing surveillance, but the absolute risk remains low. Pharmacologic therapy remains a safe and effective tool in obesity management when used with appropriate clinical judgment and individualized risk–benefit assessment. **(**Figs. 1 and 2**) (See Supplementary Material for Clinical Takeaways).**

### Partial lipodystrophy syndromes

Partial lipodystrophy Syndromes are rare disorders marked by selective loss of subcutaneous fat, severe insulin resistance, hypertriglyceridemia, and hepatic steatosis. Genetic forms, such as familial partial lipodystrophy types 1–3, typically arise from LMNA, PPARG, or other adipocyte gene mutations, with onset in adolescence or early adulthood. Acquired forms, including Barraquer–Simons syndrome, involve autoimmune mechanisms and cephalocaudal fat loss. HIV-associated lipodystrophy, linked to antiretroviral therapy, presents with variable lipoatrophy and central adiposity, often sharing similar metabolic derangements [150, 151].

In these settings, conventional Weight loss approaches are ineffective. Emerging evidence suggests that semaglutide may improve metabolic outcomes independent of Weight reduction. In a phase 2b randomized placebo-controlled trial, semaglutide 2.4 mg OW significantly reduced abdominal visceral, subcutaneous, and total body fat in people with HIV-associated lipohypertrophy without diabetes [152] (Figs. 1 and 2) (see Supplementary Material for Clinical Takeaways).

### Monogenic and syndromic obesity

Monogenic and syndromic forms of obesity, although rare, offer critical insight into the neuroendocrine regulation of appetite and body weight. These disorders are typically characterized by early-onset severe obesity, hyperphagia, and often endocrine or developmental abnormalities. Advances in molecular genetics have elucidated causative pathways, paving the way for targeted pharmacotherapies.

Congenital leptin deficiency, due to LEP mutations, exemplifies a monogenic obesity with extreme hyperphagia and rapid weight gain. Recombinant leptin therapy in these people produces marked reductions in appetite and weight, underscoring leptin’s central role in energy balance [153].

Disruption of the MC4R pathway, via biallelic mutations in POMC, PCSK1, or LEPR, impairs satiety signaling. The melanocortin receptor agonist Setmelanotide has shown efficacy in these conditions, being a selective agonist of the MC4R it mimics the action of the alpha MSH and represents a hormonal replacement therapy. In phase 3 trials, patients with POMC or LEPR deficiency experienced significant weight loss and hunger reduction with favorable safety [108]. The VENTURE trial extended these findings to children aged 2–5 years, including those with Bardet–Biedl syndrome, confirming sustained benefits in early childhood [104].

These results support early genetic screening in children with severe, early-onset obesity and the use of precision therapies like Setmelanotide in genetically confirmed MC4R-pathway disorders. While not generalizable to polygenic obesity, these targeted treatments mark a key advance in personalized obesity care (Figs. 1 and 2) (see Supplementary Material for Clinical Takeaways).

### Acquired hypothalamic obesity

Acquired hypothalamic obesity (HO) is a rare, severe form of secondary obesity resulting from structural or functional damage to the hypothalamus, typically after craniopharyngioma, suprasellar tumors, neurosurgery, or radiotherapy. This disruption impairs key energy balance pathways, including leptin–melanocortin signaling, autonomic tone, and reward-based feeding circuits. Clinically, HO presents with rapid, refractory weight gain, hyperphagia, low energy expenditure, and impaired satiety, features often resistant to conventional lifestyle-based treatment. Setmelanotide, a selective MC4R agonist, has demonstrated promising efficacy in this setting. In a recent phase 2 multicenter trial, patients with acquired HO experienced clinically meaningful weight loss and hunger reduction with good tolerability [154], supporting its use in cases with confirmed melanocortin pathway disruption. From the trial, it seems that young people have better response regarding weight loss and resolution of CVD features than adult patients, even if this was not a direct comparison between the two groups [154].

Semaglutide has also been evaluated off-label in HO. A 2024 real-world study reported moderate weight loss, improved glycemia, and acceptable tolerability in adults with hypothalamic injury, though gastrointestinal side effects and variable response were noted [155]. GLP-1 RAs may thus offer benefit in select cases, particularly where some hypothalamic function is preserved. Given the heterogeneous pathophysiology and limited therapeutic options, pharmacologic management of HO requires a highly individualized, multidisciplinary approach. Agents like Setmelanotide or GLP-1 RAs **may be integrated** alongside nutritional, behavioral, and endocrine support to optimize clinical outcomes (Figs. 1 and 2) (see Supplementary Material for Clinical Takeaways).

### Anti-obesity pharmacotherapy in pregnancy: clinical considerations

#### Pharmacologic obesity management and pregnancy

Pharmacologic treatment of obesity is contraindicated during pregnancy due to limited human safety data, known teratogenicity, and potential harm to fetal development. Current clinical guidelines from the American College of Obstetricians and Gynecologists and the Endocrine Society advise immediate discontinuation of all AOMs upon pregnancy detection. Preconception weight optimization, through lifestyle interventions and, where appropriate, short-term pharmacotherapy, is the preferred strategy (Figs. 1 and 2) (see Supplementary Material for Clinical Takeaways).

**Maternal Obesity and Pregnancy Risk**

Maternal obesity independently increases the risk of gestational diabetes, hypertensive disorders (including preeclampsia), cesarean delivery, macrosomia, and offspring metabolic dysfunction [156]. Despite these risks, no pharmacologic agent is currently approved for use during gestation.

**Safety of Pharmacologic Agents in Pregnancy**

Orlistat: Although minimally absorbed, it interferes with fat-soluble vitamin absorption (e.g., folate), raising theoretical concerns about neural tube defects. It is not recommended in pregnancy [157].

GLP-1 RAs (Semaglutide, Liraglutide): Teratogenic effects observed in animal models; agents cross the placenta and should be discontinued at least 1–2 months preconception.

Tirzepatide: No human safety data; animal studies show developmental toxicity. Strictly contraindicated in pregnancy, with similar preconception discontinuation timing as GLP-1 RAs.

Naltrexone/Bupropion: Crosses the placenta; bupropion has been linked to a small increased risk of cardiac malformations [158]. Combination use is not recommended in pregnancy.

Phentermine/Topiramate ER: Topiramate is a known teratogen, significantly increasing the risk of orofacial clefts. Contraindicated during pregnancy [159].

**Preconception and Interconception Care**

In women with obesity of reproductive age, clinical planning should address weight optimization, contraception, and drug withdrawal timelines. AOMs used before conception may improve ovulation, reduce insulin resistance, and lower the risk of gestational diabetes mellitus and hypertensive complications [160]. However, due to teratogenic risks, their use requires strict contraceptive adherence and shared decision-making aligned with reproductive goals.

### Polycystic ovary syndrome (PCOS)

PCOS and obesity share a close and interconnected relationship. PCOS is characterized by oligomenorrhea, hyperandrogenism, and the presence of polycystic ovaries on ultrasound [161]. Obesity is more prevalent among females with PCOS than in the general population; it contributes to the development of PCOS and worsens [161] its clinical features, primarily due to hyperinsulinemia resulting from insulin resistance, which increases ovarian androgen production [162, 163]. Weight loss is recommended for people with PCOS and BMI ≥ 25, as it has been shown to improve both reproductive and metabolic outcomes [161]. According to the 2023 European PCOS guidelines, AOMs and bariatric/metabolic surgery may be considered based on general population guidelines and lifestyle intervention, carefully evaluating potential benefits and side effects [161]. (Fig. 2) (See Supplementary Material for Clinical Takeaways).

**Metformin**

Metformin has a near-neutral effect on weight, with one meta-analysis finding no effect [164] and others finding small reductions in BMI, approximately of −0.77 kg/m² (95%CI: −1.26, − 0.27) compared to placebo after three months of treatment with 1500 mg/day in women with PCOS [165, 166]. Metformin is recommended in adults with PCOS and a BMI ≥ 25 to improve insulin resistance, glucose and lipid profiles, but not for weight loss [161]. It can also be used for irregular menstrual cycles, to improve pregnancy rates [161]. No benefits on hirsutism are to be expected [161]. Metformin **is not recommended** for weight loss in PCOS but may be recommended in women with BMI ≥ 25 kg/m² to improve metabolic and reproductive outcomes.

**Semaglutide**

A few RCTs evaluated semaglutide in PCOS, all at high risk of bias due to small sample size and selective outcome reporting. One study (*n* = 25) compared semaglutide 1 mg OW to placebo over 16 weeks, demonstrating greater weight loss with semaglutide (− 5.2 kg versus + 1.9 kg with placebo) [167]. Another trial (*n* = 30) using the same dosing and duration reported a similar benefit, with an estimated treatment difference of − 7.8 kg (− 7.7% in body weight) in favor of semaglutide [168]. No studies have been conducted using the weight-loss–recommended dose of 2.4 mg. Semaglutide **may be considered** in women with PCOS and obesity who are candidates for pharmacologic weight loss. Larger studies at therapeutic doses are needed to confirm reproductive and metabolic effects.

**Tirzepatide**

No PCOS-specific data are available. Tirzepatide **may be considered** in women with PCOS and obesity when substantial weight loss is the primary therapeutic target. Dedicated studies are needed to assess reproductive and metabolic outcomes.

**Liraglutide**

Liraglutide 3 mg/day led to a significantly greater reduction in body weight (− 5.7%) compared to placebo (− 1.4%), both in addition to lifestyle intervention, over 32 weeks in people with obesity and PCOS (*n* = 67) [169]. It was also superior to placebo in improving all metabolic outcomes, free androgen index and number of menstrual cycles per year, but no differences in total testosterone were observed [169]. Liraglutide **should be considered** in PCOS when weight loss and improved menstrual and metabolic parameters are desired. It may be especially useful when insulin resistance is prominent.

**Orlistat**

A randomized trial (*n* = 86) evaluated the efficacy of orlistat at a dose of 120 mg three times daily for three months, compared to placebo, both combined with lifestyle intervention [170]. The drug plus lifestyle intervention was superior to placebo plus lifestyle in achieving greater percent weight loss (− 6.37% vs. −2.27%), greater reduction in waist-to-hip ratio (0.76 vs. 0.86), and improvements in total testosterone and lipid profiles [170].However, no significant benefits were observed in other metabolic parameters [170]. Orlistat **may be considered** in PCOS when systemic exposure is a concern and modest weight loss is acceptable. Gastrointestinal side effects and adherence should be monitored.

**Naltrexone/Bupropion**

No PCOS-specific data are available.

**Phentermine/Topiramate ER**

Only one small trial has evaluated this combination in a PCOS population, comparing phentermine 7.5 mg plus topiramate 46 mg ER daily to exenatide 2 mg OW (*n* = 36 for both arms) [171]. After 24 weeks, mean weight loss with phentermine/topiramate ER in PCOS obese women was 8%, even higher than the 6.6% Weight loss reported at 56 weeks in the general population [171] However, cardiovascular safety concerns persist for this drug, particularly in people with PCOS, who are already at increased risk for cardiovascular complications compared to the general population [172]. Phentermine/topiramate **may be considered** for short-term use in women with PCOS and severe obesity, but cardiovascular risk must be carefully assessed and monitored.

## Obesity management under cost constraints

Despite the increasing availability of effective pharmacologic and surgical options, the economic burden of obesity treatment remains a major barrier to care in many healthcare systems. Cost constraints manifest at multiple levels, including a lack of reimbursement for AOMs, restricted surgical access, inadequate infrastructure, and inequitable distribution of clinical resources. In response, clinicians and policymakers must balance efficacy, affordability, and scalability to provide equitable care across socioeconomic strata. This section discusses the clinical positioning of two high-value, cost-conscious strategies: orlistat pharmacotherapy and bariatric surgery. (Figures 1 and 2) (See Supplementary Material for Clinical Takeaways).

## Obesity management under cost constraints

### QALY-Based decisions

In resource-constrained health systems, treatment choices for obesity must weigh clinical severity, patient priorities, and economic value. Quality-adjusted life years (QALYs) offer a standardized metric to evaluate cost-effectiveness by quantifying health benefits relative to cost. Interventions are considered cost-effective if their incremental cost-effectiveness ratio (ICER) is below $50,000–$100,000 per QALY in the U.S. or £20,000–£30,000 in the UK [173, 174].

#### Bariatric surgery

Roux-en-Y gastric bypass and sleeve gastrectomy remain the most cost-effective obesity treatments, especially in T2D. Surgery reduces healthcare costs within 2–5 years by lowering medication use, hospitalizations, and disability [175]. ICER estimates range from $5,000 to $35,600/QALY [176], with durable benefits including 25–35% weight loss, T2D remission, and lower cardiovascular and mortality risk [177]. Surgical capacity and need for follow-up remain barriers to broader access.

#### Orlistat

A non-systemic lipase inhibitor, orlistat offers modest weight loss (~ 3%) and T2D prevention benefits, with a 45% reduction in incident T2D in the XENDOS trial [14]. Its generic availability, safety in older adults, and minimal monitoring needs make it a cost-effective first-line choice in low-resource settings, despite frequent GI side effects and limited long-term adherence [149].

#### Semaglutide

In a cost-effectiveness analysis using a lifetime microsimulation model, oral semaglutide was associated with improved clinical outcomes compared to several antihyperglycemic treatments, including fewer MACE and higher quality-adjusted life-years (QALYs). While oral semaglutide was cost-effective compared to liraglutide (ICER = $40,100/QALY) and moderately cost-effective versus background treatment ($117,500/QALY) and sitagliptin ($145,200/QALY), it did not meet standard cost-effectiveness thresholds when compared with empagliflozin, with an estimated ICER of approximately $458,400 per QALY. This suggests that, despite clinical benefits, the high cost of oral semaglutide limits its value relative to more cost-effective alternatives like empagliflozin [178].

#### Tirzepatide

In 2024 lifetime cost-effectiveness analysis modeling U.S. adults eligible for antiobesity pharmacotherapy, tirzepatide emerged as the most clinically impactful agent among the therapies evaluated. Compared to lifestyle modification alone, tirzepatide was estimated to avert 45,609 cases of obesity, 20,854 cases of type 2 diabetes, and 10,655 cases of cardiovascular disease per 100,000 people—surpassing semaglutide and all other comparators. It also yielded the greatest incremental gain in quality-adjusted life-years (QALYs) at 0.35. However, despite these substantial health benefits, tirzepatide was not cost-effective at its current price, with an ICER of $197,023/QALY and a 0% probability of meeting standard thresholds for cost-effectiveness ($100,000–$200,000/QALY). To be considered cost-effective at the $100,000/QALY benchmark, its price would need to be reduced by 30.5%. These findings underscore the need for substantial price reductions to enable equitable access to tirzepatide as a high-efficacy antiobesity therapy [179].

**Bariatric surgery** offers the best return on investment, particularly for patients with diabetes or severe obesity. Orlistat remains a practical option where newer agents are unavailable. Advanced agents like semaglutide and tirzepatide require substantial cost reductions or innovative pricing models (e.g., value-based contracts) to support broader public reimbursement. Treatment intensity should align with disease burden, projected benefit, and fiscal sustainability in settings with limited resources.

## Gender differences in AOM and surgery response

Biological sex significantly influences the response to obesity treatments. Across major trials, women, especially premenopausal, tend to experience greater relative weight loss with GLP-1 RAs and dual incretin therapies such as tirzepatide [2, 3]. This may reflect differences in leptin levels, estrogen-regulated satiety pathways, and better adherence to lifestyle interventions.

Recent pharmacogenetic findings suggest a sex-specific interaction with GLP-1 receptor variants. A GLP-1R polymorphism was linked to enhanced semaglutide response in females, hinting at sex-linked differences in receptor sensitivity [180].

Men, by contrast, often present with higher visceral adiposity and sarcopenic risk, which may attenuate weight loss and predispose to muscle loss during high-efficacy pharmacotherapy [181]. This may lead to fatigue or functional decline without appropriate resistance training and protein support.

Adverse effects also differ: women report higher rates of gastrointestinal intolerance (nausea, vomiting, cholelithiasis) with incretin-based therapies, likely due to estrogen’s effects on biliary motility [182]. These symptoms can impair adherence, particularly in people with low motivation or disordered eating.

Sex differences extend to bariatric surgery: women achieve greater total body weight loss and glycemic improvements, while men face higher postoperative risk and lower rates of T2D remission [183].

Despite these observations, most obesity trials are not powered for sex-specific analyses, and subgroup findings remain exploratory. Clinical strategies should consider sex-informed modifications, e.g., slower drug titration in women to reduce GI side effects, or tailored sarcopenia prevention in men. Future trials should integrate sex-stratified endpoints to enhance therapeutic precision and equity in obesity care. (See Supplementary Material for Clinical Takeaways).

## The non-responders: Understanding variability in obesity treatment outcomes

Despite the overall efficacy of AOMs and bariatric surgery, a significant proportion of people, referred to as non-responders, fail to achieve or maintain clinically meaningful weight loss. Early identification and tailored treatment adaptation are crucial for optimizing outcomes.

In AOM trials, non-response is typically defined as < 5% total body Weight loss at 12–16 weeks. In STEP 1, ~ 13.6% of participants on semaglutide 2.4 mg did not meet this threshold despite adherence at week 68 [3]. Similarly, in SURMOUNT-1, non-response rates for tirzepatide were 15% (5 mg), 11% (10 mg), and 9% (15 mg) at 72 weeks [2]. Real-world non-response rates may be higher due to variations in adherence and population characteristics.

Early weight loss trajectory strongly predicts long-term outcomes. Failing to lose ≥ 0.5% body Weight per week in the first month is associated with suboptimal long-term results. In a 2025 RCT, early non-responders to behavioral therapy who received phentermine 15 mg/day lost 3.1% more Weight at 24 weeks than those continuing therapy with placebo (5.9% vs. 2.8%; *P* = 0.003) [184].

In bariatric surgery, up to one-third of patients may exhibit suboptimal outcomes (< 50% excess Weight loss at 1 year or weight regain). Predictors include early-onset obesity, male sex, preoperative weight gain, T2D, poor behavioral adherence, and psychiatric comorbidity [185]. Post-surgical strategies include structured lifestyle re-engagement, adjunctive pharmacotherapy (e.g., GLP-1 RAs), and revisional surgery when appropriate [186]. Ultimately, the proactive management of non-responsiveness through timely escalation, combination therapies, behavioral reinforcement, or surgical referral should be integrated into clinical pathways. Personalized strategies tailored to phenotype, early response, and psychosocial context remain central to effective, sustainable obesity care.

## AOMs: combination strategies

As monotherapy with GLP-1 RAs or tirzepatide increasingly achieves double-digit weight loss, combination pharmacotherapy is emerging as a promising frontier in obesity treatment. In selected patients, such as partial responders, those with drug intolerance, behavioral phenotypes, or complex cardiometabolic profiles, rational co-therapy may enhance both efficacy and personalization. These strategies, tailored by clinical phenotype, comorbidities, tolerability, and cost, will require integration into future therapeutic algorithms, including thresholds for monotherapy optimization and criteria for escalation. Below, we summarize the most advanced and evidence-supported combination approaches currently under evaluation.

### GLP-1 RA + Naltrexone/Bupropion

Combining GLP-1 RAs with naltrexone/bupropion targets both homeostatic and hedonic drivers of obesity. GLP-1 analogues enhance satiety via hypothalamic and brainstem circuits, while naltrexone bupropion reduces reward-driven eating and impulsivity through dopaminergic-opioidergic modulation. A real-world study demonstrated that GLP-1 analogues are an effective treatment for weight loss, and the addition of bupropion/naltrexone is associated with greater weight loss including in patients who are initially non-responsive to GLP-1 analogues [187]. Nonetheless, overlapping GI and neuropsychiatric side effects necessitate slow titration and careful monitoring.

### GLP-1 RA + Orlistat

In patients with limited access to high-dose GLP-1 RA or intolerant to full titration, combining liraglutide with orlistat may provide modest additional weight loss via peripheral fat absorption blockade. An open-label trial showed greater efficacy than orlistat alone, though gains over liraglutide monotherapy were marginal [188]. This strategy may be considered in cost-constrained or older populations.

### Semaglutide + Cagrilintide

This is the most advanced fixed-dose combination in development. Cagrilintide, a long-acting amylin analog, enhances satiety and delays gastric emptying. In a 32-week Phase II trial, semaglutide plus cagrilintide achieved up to 17.1% placebo-subtracted weight loss, exceeding that of semaglutide alone [189]. The ongoing REDEFINE Phase III program may position this pairing as a non-surgical alternative for patients targeting > 15–20% weight loss [78, 190].

### Tirzepatide + Future agents or triple agonists

Tirzepatide monotherapy induces Weight loss up to 22.5%, reducing the need for adjuncts in most patients. However, in partial responders or those unable to escalate dose, future strategies may involve agents such as peripherally acting cannabinoid type 1 receptor antagonists, FGF21 analogs, amylin mimetics, or emerging triple agonists like retatrutide, which target GLP-1, GIP, and glucagon receptors simultaneously. Early data suggest potential for non-surgical > 25% weight loss, though clinical integration awaits Phase III results. (See Supplementary Material for Clinical Takeaways).

**GLP-1 RAs/dual GIP/GLP-1 RAs + ketogenic diet**: both liraglutide and tirzepatide have been associated with the ketogenic diet with good results: the association with tirzepatide leading to more significant fat mass loss and muscle mass preservation [191], that with low dose liraglutide (up to 1.8 mg) leading to more significant weight loss and marked insulin resistance improvement [192].

## Bariatric surgery and aoms: combination strategies for weight regain and insufficient weight loss

Although bariatric surgery is the most effective intervention for sustained Weight loss and metabolic improvement in patients with severe obesity, up to one-third of people experience inadequate Weight loss or significant weight regain within 2–5 years [193]. This has prompted the integration of pharmacologic adjuncts into post-surgical care pathways, particularly for those with ongoing obesity-related comorbidities or a high risk of relapse.

GLP-1 RAs particularly liraglutide and semaglutide are the most studied agents in this Context. In an extensive retrospective study, semaglutide 2.4 mg OW was found to be more effective than liraglutide 3.0 mg daily in treating weight regain after bariatric surgery, achieving greater total body weight reduction and improved tolerability [194]. These findings support the superiority of OW agents in this context and justify their consideration as first-line pharmacologic options when weight recurrence occurs.

Earlier studies have demonstrated the benefit of liraglutide in similar populations. In real-world data, liraglutide 3.0 mg led to meaningful weight loss in patients with prior bariatric procedures, particularly when initiated in the early phase of weight regain [195, 196]. These effects were more pronounced in patients with behavioral adherence and preserved gastrointestinal anatomy, suggesting a potential role for phenotype-guided prescribing.

More recently, tirzepatide has emerged as a promising therapeutic escalation for patients with suboptimal response after surgery. In a 24-week observational study, tirzepatide resulted in clinically significant weight loss and favorable body composition changes, including reduction of visceral adiposity while largely preserving lean mass [197]. These findings suggest that dual incretin agonists may reverse weight recurrence and enhance metabolic quality of weight loss post-surgery. (Fig. 1) (See Supplementary Material for Clinical Takeaways).

## Safety and monitoring in Anti-Obesity pharmacotherapy

Pharmacologic obesity treatment requires individualized safety assessment and vigilant monitoring to ensure benefit outweighs risk. As AOMs are prescribed across a range of ages, metabolic profiles, and comorbid conditions, clinicians must consider tolerability, contraindications, and the potential for drug–drug interactions. Special attention should be paid to early adverse effects, long-term safety signals, and population-specific vulnerabilities. **(See Supplementary Material for Clinical Takeaways).**

**GLP-1 RAs (Semaglutide**,** Liraglutide)**

GLP-1 RAs are generally well tolerated, though gastrointestinal side effects, especially nausea, vomiting, and diarrhea, are common, particularly during dose escalation. In STEP 1, up to 74% of participants reported nausea with semaglutide 2.4 mg, yet discontinuation for GI reasons was relatively low (4.5%) [3]. Long-term trials like LEADER confirmed cardiovascular and renal safety for liraglutide in T2D, with no increased risk of pancreatitis or thyroid malignancy [34].

Suggested monitoring:

- Renal function, particularly in patients with CKD or persistent vomiting.
- Gallbladder symptoms, especially with rapid weight loss.
- Electrolytes and glycemia, in patients on concomitant glucose-lowering therapy.

**Dual Incretin Agonist (Tirzepatide)**

Tirzepatide offers superior weight loss efficacy with a safety profile similar to GLP-1 RAs. In SURMOUNT-5, GI side effects were comparable to semaglutide, although slightly more frequent at higher tirzepatide doses. Discontinuation for adverse events was 6.1% for tirzepatide versus 8.0% for semaglutide [76]. While long-term safety is still under investigation, current data suggest favorable cardiometabolic outcomes in T2D populations.

Special considerations: in older or sarcopenic adults, weight loss should be accompanied by assessment of lean mass and functional status.

**Naltrexone/Bupropion**

This centrally acting combination targets both homeostatic and hedonic pathways. Adverse events include nausea, headache, insomnia, and modest increases in heart rate and blood pressure. Contraindications include uncontrolled hypertension, seizure history, and ongoing opioid use. Psychiatric monitoring is essential, particularly in patients with mood disorders. Although the LIGHT CVOT was halted early [15], annual cardiovascular risk evaluation is advised.

**Orlistat**

A peripherally acting lipase inhibitor, orlistat avoids systemic exposure but causes fat malabsorption-related symptoms such as steatorrhea and fecal urgency, especially with high-fat diets. Rare risks include fat-soluble vitamin deficiency, oxalate nephropathy, and idiosyncratic hepatotoxicity [14, 149].

Monitoring:

- Liver enzymes and urinalysis with prolonged use.
- Fat-soluble vitamin levels if not supplemented.

**Phentermine/Topiramate ER**.

This sympathomimetic–anticonvulsant combination requires careful monitoring due to neurologic and cardiovascular effects. Common side effects include paresthesia, dry mouth, mood changes, and cognitive slowing. Topiramate is teratogenic; pregnancy must be excluded and effective contraception ensured in women of childbearing potential [16, 60].

Monitoring:

- Blood pressure, heart rate.
- Mood and cognitive symptoms.
- Contraceptive adherence.

Avoid in: recent cardiovascular events, unstable psychiatric conditions.

Special Populations.

- Older Adults: Prioritize agents with benign CNS profiles (e.g., orlistat, low-dose liraglutide); monitor for sarcopenia and interactions.
- Adolescents (≥ 12 years): Semaglutide and liraglutide are approved. Monitor growth, GI tolerance, and psychosocial health [109, 113].
- Renal Impairment: Avoid agents that promote volume depletion.
- Psychiatric Comorbidity: Use caution with medications affecting mood or cognition; close follow-up is essential.

## Discussion

The paradigm of obesity pharmacotherapy is undergoing a major shift from a weight-centric, BMI-driven approach to a precision strategy that integrates phenotypic and complication-oriented profiling. This review consolidates emerging evidence supporting the view that AOMs exert heterogeneous effects based on underlying metabolic, behavioral, and organ-specific characteristics. GLP-1 RAs, dual GIP/GLP-1 RAs (e.g., tirzepatide), and next-generation triple agonists such as retatrutide are not simply tools for weight loss they are disease-modifying agents capable of impacting cardiovascular, renal, hepatic, mechanical and behavioral endpoints, provided they are matched appropriately to patient phenotype [198]. In patients who already present with obesity-related complications, pharmacologic therapy should be considered as a first-line option alongside lifestyle intervention, without postponing treatment until lifestyle approaches have failed. This principle reflects the need for timely intervention to modify disease trajectories and mitigate organ damage.

Among people with established ASCVD, semaglutide 2.4 mg has demonstrated a 20% relative risk reduction in MACE, establishing it as the only AOM with proven benefit in secondary prevention [4]. In patients with HFpEF and obesity, both semaglutide and tirzepatide improve NYHA class, physical function, and quality of life independent of glycemic control, suggesting a glucose and weight-independent mechanism linked to anti-inflammatory and hemodynamic effects [5, 11].

Beyond cardiovascular endpoints, the renoprotective effects of these agents are becoming increasingly evident. In the FLOW trial, semaglutide slowed eGFR decline and reduced the incidence of renal events, including progression to kidney failure, in people with diabetic CKD [199]. Importantly, these benefits were observed even in patients with an eGFR as low as 30 mL/min/1.73 m², highlighting the broad applicability of incretin-based agents in renal risk reduction.

Liver-related complications are also amenable to pharmacologic reversal. In MASLD and MASH, semaglutide induces histological improvement, including MASH resolution and fibrosis regression, with > 60% of patients achieving resolution in F2–F3 stages. These results mirror improvements in MRI-PDFF and liver enzymes, confirming their utility in the hepatic phenotype. This supports the growing consensus that GLP-1–based pharmacotherapy represents a foundational treatment in the management of metabolic liver disease, even prior to the availability of dedicated MASH drugs [54].

A critical frontier is the management of people without overt complications. Although traditional guidelines prioritize treatment in those with comorbidities, mounting evidence shows that early pharmacologic intervention in high-risk but complication-free people can be preventive. For example, tirzepatide has demonstrated up to 94% relative risk reduction in progression to T2D among prediabetic people, while semaglutide delays onset of dysglycemia, cardiovascular events, and renal decline in metabolically healthy patients with obesity, reverting to normoglycemia by 81% vs. 14% (placebo) [22]. These findings challenge the binary distinction between primary and secondary prevention and support a broader application of AOMs for risk mitigation.

Behavioral phenotypes further refine treatment personalization. People with BED, emotional dysregulation, or compulsive food-related behaviors often exhibit poor adherence and suboptimal response to generic interventions. GLP-1 RAs and naltrexone/bupropion have shown efficacy in attenuating hedonic drive through hypothalamic and mesolimbic modulation, offering therapeutic tools beyond caloric restriction [200]. However, trial representation remains low, and real-world validation is needed.

Age-specific considerations are also paramount. Adolescents respond well to semaglutide and liraglutide, with marked reductions in BMI and cardiometabolic risk factors. In contrast, pharmacologic strategy in older adults must balance efficacy with safety: slower-acting agents such as liraglutide, or gut-restricted compounds like orlistat, may be preferred to avoid excessive weight loss, sarcopenia, or nutritional deficiencies. Sarcopenic obesity represents a particularly complex phenotype where the goal is not weight loss per se, but improved physical function. Liraglutide combined with resistance training and high-protein intake has demonstrated preservation of lean mass and strength, making it a suitable option in this setting [201].

Importantly, this framework acknowledges that most patients present with overlapping phenotypes. For instance, a postmenopausal woman with prediabetes, MASLD, and depressive symptoms may fall under multiple therapeutic targets. Clinicians must weigh dominant pathophysiologic drivers while avoiding contraindicated agents. In such cases, the pharmacologic decision should be guided by dominant morbidity (e.g., liver fibrosis or binge eating), expected benefits, and safety profile, ideally informed by shared decision-making.

### Strengths and limitations

The primary strength of this review lies in its multidimensional framework, integrating high-quality randomized controlled trials, real-world evidence, regulatory documents, and expert consensus to support a phenotype- and complication-oriented treatment strategy for obesity. By organizing evidence across age groups, organ-specific phenotypes, and behavioral traits, this review more closely mirrors clinical complexity than traditional BMI-based guidelines. Moreover, emphasis on cost-effectiveness and safety across diverse populations enhances clinical utility and implementation potential.

However, several limitations should be acknowledged. First, the absence of direct head-to-head trials between different AOMs limits comparative conclusions. Second, behavioral and psychosocial phenotypes remain underrepresented in major trials, constraining generalizability. Third, cost-effectiveness models are highly context-specific and may not extrapolate across healthcare systems.

## Conclusion

The clinical utility of AOMs now extends far beyond weight reduction. By targeting phenotype and complication clusters, ranging from cardiovascular to hepatic and behavioral axes, clinicians can tailor pharmacologic regimens to achieve disease modification, symptom relief, and prevention. In people presenting with obesity-related complications, pharmacologic treatment should be considered as first-line, in conjunction with lifestyle therapy, to address disease burden and avoid therapeutic inertia promptly. As machine learning models and non-invasive biomarkers mature, they will increasingly guide precision medicine in obesity care, answering the following critical questions: which patient, which drug, and at what time. The current evidence base justifies a proactive, complication-oriented deployment of pharmacotherapy to reduce long-term obesity burden and improve quality-adjusted survival across diverse populations.

## Supplementary Information

Below is the link to the electronic supplementary material.

Supplementary Material 1

## Data Availability

This narrative review is based on published sources; no new data were generated or analyzed.

## Abbreviations

AAP: American Academy of Pediatrics
AC: Abdominal Circumference
ADA: American Diabetes Association
ALT: Alanine Aminotransferase
AST: Aspartate Aminotransferase
ASCVD: Atherosclerotic Cardiovascular Disease
AOM: Anti-Obesity Medication
AOMs: Anti-Obesity Medications
BMD: Bone Mineral Density
BMI: Body Mass Index
BP: Blood Pressure
CPCG: Canadian Clinical Practice Guidelines
CKD: Chronic Kidney Disease
CRP: C-Reactive Protein
DBP: Diastolic Blood Pressure
eGFR: Estimated Glomerular Filtration Rate
EASO: European Association for the Study of Obesity
FFM: Fat-Free Mass
GGT: Gamma-Glutamyl Transferase
GI: Gastrointestinal
GLP-1: Glucagon-like Peptide-1
GIP: Glucose-dependent Insulinotropic Polypeptide
HbA1c: Hemoglobin A1c
HF: Heart Failure
HFpEF: Heart Failure with Preserved Ejection Fraction
HOMA-IR: HOmeostatic Model Assessment for Insulin Resistance
HRQoL: Health-Related Quality of Life
ICER: incremental cost-effectiveness ratio
LDL: Low-Density Lipoprotein
MASLD: Metabolic Dysfunction-Associated Steatotic Liver Disease
MASH: Metabolic Dysfunction-Associated Steatohepatitis
MACE: Major Adverse Cardiovascular Events
MHO: Metabolically Healthy Obese
MUO: Metabolically Unhealthy Obese
PA: Physical Activity
PCOS: Polycystic Ovary Syndrome
QALYs: Quality-adjusted life years
QoL: Quality of Life
RCT: Randomized Controlled Trial
RMR: Resting Metabolic Rate
SBP: Systolic Blood Pressure
T1D: Type 1 Diabetes
T2D: Type 2 Diabetes
WC: Waist Circumference
WOMAC: Western Ontario and McMaster Universities Osteoarthritis Index
