# Supplementary material for: Precision obesity medicine: A phenotype-guided framework for pharmacologic therapy across the lifespan
Source: J Endocrinol Invest. 2025 Nov 10;48(12):2761–98. doi: 10.1007/s40618-025-02700-7 (PMC12640352; doi:10.1007/s40618-025-02700-7)
Supplement: Supplementary file 1 — Supplementary Material 1 [file 40618_2025_2700_MOESM1_ESM.docx]

| **Clinical Takeaways box: ASCVD and HF in Obesity**  • Cardiovascular disease is the primary cause of mortality in obesity, even without diabetes. AOMs that reduce adiposity and inflammation may improve outcomes, especially in ASCVD and HFpEF phenotypes.  • **Semaglutide**: In SELECT, semaglutide 2.4 mg weekly reduced MACE by 20% (HR 0.80) and all-cause mortality by 19%. In STEP-HFpEF, in 529 participants, it improved KCCQ (+7.8 points), 6MWD (+20.3 m), CRP (−43.5%), and body weight (−13.3%); HF event rate was lower (HR 0.08; exploratory). Semaglutide **should be preferred** in people with obesity and established ASCVD or HFpEF, even in the absence of diabetes, based on robust CV benefit.  • **Tirzepatide**: In SUMMIT, reduced composite CV death or HF hospitalization (HR 0.62), driven by reduced HF hospitalizations (HR 0.54); improved KCCQ (+6.9 points) and 6MWD (+18.3 m). SURMOUNT-1 post hoc analyses showed improvements in ASCVD risk scores, BP, lipids, and inflammation. Tirzepatide **should be preferred** for people with obesity and HFpEF; CV benefit appears promising but awaits dedicated CVOT data.  • **Liraglutide**: In patients with obesity and prediabetes, improved BP and lipids over 3 years. In post hoc SCALE analyses, liraglutide 3.0 mg had a neutral CV safety profile, but no data support use in HF prevention. Liraglutide **may be considered** in people with obesity and elevated CV risk who are not candidates for more potent agents; not recommended specifically for HF.  • **Orlistat**: In XENDOS, led to modest weight loss and BP improvement, with a neutral effect on CV outcomes. Orlistat **may be considered** when centrally acting AOMs are contraindicated, though long-term efficacy is limited.  • **Naltrexone/Bupropion**: LIGHT trial was terminated early; CV safety is unproven. Sympathomimetic effects (↑HR, BP) raise concern. This combination **is not recommended** for people with ASCVD or HF due to potential hemodynamic stress and lack of outcome data, especially when alternatives with proven benefit are available.  • **Phentermine/Topiramate**: No CVOTs available. EQUIP showed modest improvements in BP and lipids, but phentermine’s effects on HR/BP raise safety concerns. This combination **is not recommended** high-risk people with ASCVD or HF, especially when alternatives with proven benefit are available. |
| --- |

| **Clinical Takeaways box: CKD in Obesity**  • CKD increases cardiometabolic risk and complicates AOM use due to altered clearance and adverse effect potential. AOM selection should be guided by renal outcomes, tolerability, and stage of CKD.  • **Semaglutide**: Reduced renal composite endpoint by 22% (SELECT: ≥50% eGFR decline, macroalbuminuria, RRT), observational data support safety up to 1 mg OW in dialysis patients. Semaglutide **should be preferred** as first-line AOM in people with obesity and CKD, including non-diabetic populations, based on renal and CV outcome data.  • **Tirzepatide**: Improved eGFR (creatinine and cystatin C-based), reduced UACR in SUMMIT (n = 731), and SURMOUNT-1 post hoc analyses, no renal CVOTs to date. Tirzepatide **may be considered** when semaglutide is not tolerated, although renal benefits remain to be confirmed in dedicated trials (TREASURE-CKD, ClinicalTrials.gov ID NCT05536804).  • **Orlistat**: Preserved renal function in a 2-year study in CKD stages 3–5; population data show lower CKD incidence (HR 0.78); oxalate nephropathy risk requires caution. Orlistat **may be considered** in people with moderate-to-severe CKD when incretin therapies are contraindicated, with close renal monitoring.  • **Naltrexone/Bupropion**: No renal outcome data; sympathomimetic effects (↑HR, ↑BP) raise concern for hemodynamic renal injury. This combination **is not recommended** people with moderate-to-severe CKD (eGFR <60 mL/min/1.73 m²), given uncertain benefit and safety concerns.  • **Phentermine/Topiramate**: No renal CVOTs; topiramate undergoes renal excretion and may cause acidosis or nephrolithiasis; phentermine increases BP. This combination **is not recommended** people with eGFR <60 or with nephrolithiasis risk, due to potential for harm and lack of renal outcome data. |
| --- |

| **Clinical Takeaways box: Prediabetes in Obesity**  • Prediabetes is a high-risk, reversible state driven by excess adiposity and insulin resistance; AOMs that achieve ≥10% weight loss can restore normoglycemia and prevent T2D.  • **Semaglutide**: In STEP 1, semaglutide 2.4 mg weekly induced 10–15% weight loss and normoglycemia in 84.1% of participants with prediabetes. STEP 10 (n = 207) confirmed similar glycemic and weight benefits. Semaglutide **should be preferred** in people with obesity and prediabetes, based on high rates of diabetes prevention and metabolic improvement.  • **Tirzepatide**: In SURMOUNT-1, 15 mg weekly led to 22.5% weight loss and a 94% reduction in diabetes progression over 72 weeks. In a dedicated prevention trial (n = 2539), tirzepatide reduced T2D incidence from 13.3% to 1.3% (HR 0.07). Tirzepatide **should be preferred** in people with obesity and prediabetes, when greater weight loss is needed.  • **Metformin**: In the DPP, reduced T2D incidence by 31% over 2.8 years; effect stronger in younger and more overweight people. Weight loss modest (~2–3 kg). Metformin **may be considered** in prediabetes, especially when cost or tolerability is a concern; less effective for weight-driven glycemic reversal.  • **Acarbose**: in the STOP -NIDDM trial acarbose lowers weight by ~0.5–0.8 kg and delays glucose absorption; modest glycemic effect with limited use in obesity. Acarbose **may be considered adjunctively** in select people with postprandial hyperglycemia, though limited by GI side effects and minimal weight benefit.  • **Orlistat**: In XENDOS, reduced diabetes incidence by 45% over 4 years, with ~2.9 kg greater weight loss vs placebo. Adherence limited by GI effects and fat intake restrictions. Orlistat **may be considered** when incretin-based therapies are contraindicated, particularly in early-stage dysglycemia.  • **Phentermine/Topiramate ER**: In CONQUER post hoc analysis reduced T2D risk by 48–78%, depending on dose; associated with up to 10.9% weight loss and improved insulin sensitivity. This combination **may be considered** in prediabetes requiring more potent weight loss, pending psychiatric and CV risk profile.  • **Naltrexone/Bupropion**: Not formally tested in diabetes prevention; COR trials (COR-I ; COR-II) showed modest improvements in fasting glucose and HOMA-IR. Sympathomimetic activity may limit use in cardiometabolic disease. This agent **may be considered** in people with obesity, emotional eating, and mild dysglycemia without significant CV risk. |
| --- |

| **Clinical Takeaways box: T2D and Obesity** • Over 80% of people with T2D have overweight or obesity. AOMs now play a central role in improving both glycemia and weight. A ≥5–15% weight loss leads to substantial reductions in HbA1c, insulin needs, and cardiovascular complications. Incretin-based AOMs in T2D • **Semaglutide**: In STEP 2, semaglutide 2.4 mg weekly resulted in 9.6% mean weight loss and ~1.6% HbA1c reduction in patients with T2D. Improvements in insulin sensitivity were also observed. **Should be preferred** in people with T2D and obesity requiring combined weight and glycemic control. **Should be prioritized** in patients with established ASCVD (secondary prevention), based on MACE reduction observed in the SUSTAIN 6.  • **Tirzepatide**: SURPASS trials showed 11–15% weight loss and HbA1c reduction up to −2.08%. In SURMOUNT-2, 15 mg led to 14.7% weight loss over 72 weeks, with 84% achieving HbA1c <7.0% and 49% <5.7%. **Should be preferred** in people with T2D and obesity when both HbA1c and weight burden are high, or when semaglutide is insufficient. It currently offers the most potent dual effect on glycemia and weight.  • **Liraglutide**: At 3.0 mg, ~6% weight loss and 1.3% HbA1c reduction (SCALE-Diabetes); at 1.8 mg, reduced MACE by 13% and CV mortality by 22% (LEADER). **May be considered** in patients with ASCVD or when daily injection is preferred; a valid alternative if weekly agents are not tolerated. Non-Incretin AOMs in T2D • **Orlistat**: Weight loss ~3% and HbA1c reductions of 0.3–0.5%; GI side effects are common. **May be considered** in people unable to use systemic AOMs, but adherence and efficacy are limited.  • **Phentermine/Topiramate ER**: In pooled T2D analyses, led to up to 9.4% weight loss and −0.4% HbA1c reduction over 56 weeks. **May be considered** in those needing potent weight loss and without CV/psychiatric contraindications.  • **Naltrexone/Bupropion**: In COR-Diabetes, −5% weight and −0.6% HbA1c; less potent than GLP-1 RAs. **May be considered** in selected people with emotional eating and controlled BP/psychiatric history. |
| --- |

| **Clinical Takeaways box: Autoimmune Diabetes and Obesity**  • Obesity in autoimmune diabetes (e.g., T1D) worsens insulin resistance, increases insulin needs, and contributes to hepatic steatosis, inflammation, and vascular/skeletal complications. • Weight gain from exogenous insulin can perpetuate dysglycemia and cardiometabolic risk. Carefully selected AOMs **may reduce insulin dose and improve control**, even without β-cell recovery. Incretin-Based Therapies (Off-Label Use) • **Semaglutide**: In a 12-month real-world study of 50 adults with T1D and obesity, semaglutide led to an average weight loss of −8.8 kg and HbA1c reduction without increasing hypoglycemia or ketoacidosis risk. When combined with automated insulin delivery, semaglutide improved both weight and glucose profiles. **May be considered** in selected people with T1D and obesity to reduce insulin requirement and body weight, with appropriate monitoring.  • **Tirzepatide**: In a retrospective cohort (n=51), tirzepatide led to 8.5% weight loss, 0.9% HbA1c reduction, and a 31.6% reduction in insulin dose over 8 months. No increase in hypoglycemia; main side effects were GI-related. Pooled data suggest up to 21.4% total body weight loss and 0.68% HbA1c reduction. **May be considered** in selected T1D patients with marked obesity and insulin resistance, though its off-label use requires close glycemic monitoring.  **Clinical Note**: • These findings are based on **non-randomized, real-world data**, and incretin use in autoimmune diabetes remains **off-label**. • If used, therapy should be **restricted to experienced centers** and to patients with **high insulin needs, obesity, and preserved awareness of hypoglycemia**, with continuous glucose monitoring and ketoacidosis vigilance.  Let me know if you want an additional section on bariatric surgery in T1D or an expert consensus box summarizing criteria for AOM off-label use in autoimmune diabetes. |
| --- |

| **Clinical Takeaways box: MASLD / MASH in Obesity** • MASLD (formerly NAFLD) is driven by excess adiposity and insulin resistance; its progressive form, MASH (formerly NASH), is associated with ballooning, inflammation, and fibrosis. • Sustained ≥10% weight loss is the cornerstone of MASH treatment but is rarely achieved with lifestyle alone. Incretin-based therapies are the most promising pharmacologic option for disease modification. Incretin-Based Therapies • **Semaglutide**: – Phase 2 (0.4 mg/day, 72 weeks): MASH resolution in 59% vs 17% with placebo; no significant fibrosis improvement. – ESSENCE Phase 3 (2.4 mg/week): 62.9% MASH resolution, 36.8% fibrosis improvement; 32.7% achieved both endpoints. Semaglutide **should be preferred** in people with biopsy-proven MASH or MASLD with fibrosis and concurrent T2D or metabolic syndrome. Although not yet approved, off-label use is **common and supported by robust RCT data**.  • **Tirzepatide**: – Phase 2b (52 weeks): MASH resolution in 44–62% (dose-dependent); fibrosis improvement in 51–55%; GI side effects mild/moderate. Tirzepatide **may be considered** in people with MASH and high weight burden or insulin resistance, especially when semaglutide is not tolerated.  • **Liraglutide**: – LEAN study: 39% MASH resolution vs 9% placebo; reduced fibrosis progression and improved liver enzymes. Liraglutide **may be considered** in early MASLD or if higher-potency incretins are not tolerated. Effect less robust in advanced fibrosis. Non-Incretin Therapies • **Orlistat**: – Improves steatosis, ALT, and insulin sensitivity; meta-analysis confirmed modest liver fat reduction. – No effect on fibrosis; no biopsy-controlled MASH data. Orlistat **may be considered** in early MASLD when incretin-based therapies are contraindicated; monitor for GI intolerance.  • **Naltrexone/Bupropion**: – Limited indirect evidence of hepatic fat/transaminase reduction; no histologic data. **may be considered** in MASLD/MASH; may be cautiously considered in patients with mild steatosis and emotional eating, avoiding use in hepatic dysfunction.  • **Phentermine/Topiramate ER**: – Weight loss 10–12%; ALT reduction and improved insulin sensitivity reported; no biopsy validation. This combination **may be considered** in patients with visceral obesity and MASLD when incretins are unsuitable, though lacks histologic efficacy data. |
| --- |

| **Clinical Takeaways box: Osteoarthritis (OA) and Obesity**  • **Semaglutide**: – In STEP 9 (n=407 with obesity and knee OA), semaglutide 2.4 mg weekly led to −13.7% weight loss, significantly reduced knee pain (WOMAC −41.7 vs. −27.5), and improved function (SF-36 +12.0 vs. +6.5) vs placebo over 68 weeks (all P<0.001). Semaglutide **should be preferred** in people with obesity and knee OA who meet criteria for pharmacologic weight management, particularly when pain and function impair quality of life.  • **Tirzepatide**: – No OA-specific outcomes yet. However, STOP KNEE-OA (phase 4) is ongoing to evaluate knee structure and joint replacement delay. Tirzepatide achieves ~21% weight loss, which is likely to relieve joint symptoms indirectly. Tirzepatide **may be considered** in patients with obesity and OA where greater weight loss is required, pending dedicated OA outcomes (STOP KNEE-OA, clinicaltrial.gov n NCT01985165).  • **Orlistat**: – Associated with OA symptom improvement when >10% weight loss is achieved, though not formally studied for OA endpoints. Orlistat **may be considered** when other AOMs are contraindicated or not tolerated, especially in early OA, with adherence counseling.  • **Naltrexone/Bupropion**: – No clinical data in OA; possible anti-inflammatory and appetite-regulatory effects hypothesized. **may be considered** for OA symptom improvement; use only if indicated for other behavioral phenotypes and with caution.  • **Phentermine/Topiramate ER**: – Induces 10–12% weight loss; no specific OA trials available. This combination **may be considered** in patients with obesity and OA who are not candidates for incretin therapies, based on extrapolated benefits from weight reduction. |
| --- |

| **Clinical Takeaways box: Obstructive Sleep Apnea (OSA) and Obesity** • OSA affects 40–60% of people with obesity, driven by pharyngeal adiposity, visceral fat, reduced airway tone, and systemic inflammation. • OSA contributes to cardiometabolic risk (HTN, T2D, MASLD, HFpEF, arrhythmia), and increases all-cause and CV mortality. • Sustained weight loss is the cornerstone of therapy; AOMs capable of ≥10–15% weight loss and central respiratory modulation may improve OSA severity. Anti-Obesity Medications in OSA • **Tirzepatide**: – First FDA-approved pharmacologic therapy for moderate-to-severe OSA in adults with obesity (Dec 2024). – In SURMOUNT-OSA-1/2 (n=469): reduced AHI by −25.3 and −29.3 events/h vs −5.3 and −5.5 with placebo (P<0.001). – 62.8% AHI reduction with tirzepatide 15 mg; 18.1% weight loss; improved oxygenation, BP, and daytime sleepiness. **Should be preferred** in people with obesity and moderate-to-severe OSA, particularly when PAP therapy is poorly tolerated or insufficient.  • **Liraglutide**: – SCALE Sleep Apnea trial (n=359): −12.2 AHI events/h with liraglutide 3.0 mg vs −6.1 placebo (P=0.015); 5.7% vs 1.6% weight loss. – Modest improvements in oxygenation and blood pressure noted. **May be considered** in patients with obesity and mild-to-moderate OSA when tirzepatide is contraindicated or unavailable.  • **Phentermine/Topiramate ER**: – In a 28-week RCT (n=45), mid/high-dose reduced AHI by −31.5 events/h vs −16.6 placebo (P=0.0084); also improved oxygenation and sleep quality. **May be considered** in patients with OSA and obesity where rapid weight loss is indicated, and other agents are not suitable, but caution is warranted in patients with CV risk.  • **Orlistat and Naltrexone/Bupropion**: – Promote weight loss but lack direct evidence for AHI reduction. **may be considered** for OSA-specific treatment; their role is limited to indirect benefit via weight loss. |
| --- |

| **Clinical Takeaways box: Weight Loss Milestones and Treatment Stratification** • Clinical benefits of weight loss occur on a continuum, with distinct thresholds linked to remission or improvement in T2D, MASLD, CV risk, and functional outcomes. • GLP-1 RAs and GIP/GLP-1 agonists offer benefits beyond weight loss—reducing inflammation, improving endothelial function, and enhancing lipid metabolism. • Weight targets should be personalized based on disease severity, phenotype, age, and patient goals. Milestone: 5–10% Weight Loss Associated with improvements in glycemia, BP, and lipids.  · **Orlistat**: ~2.9 kg placebo-adjusted loss (XENDOS). *It may be considered* when systemic agents are contraindicated.  · **Liraglutide 3.0 mg**: ~8% loss (SCALE Obesity). *It Should be preferred* in early disease or when once-daily administration is acceptable.  · **Naltrexone/Bupropion**: ~5% loss; appetite/craving reduction. *May be considered* in emotional eating phenotypes with low cardiometabolic risk.  · **Phentermine/Topiramate ER (low-mid dose)**: ~7.8% loss (CONQUER). *May be considered* when CV risks are controlled. Milestone: 10–20% Weight Loss Associated with remission of T2D and MASLD, OA relief, and OSA improvement.  · **Semaglutide 2.4 mg**: 14.9% loss (STEP 1). *It should be preferred for ASCVD, HF, OA, T2D and MASLD/MASH.*  . **Tirzepatide 5–10 mg**: ~16% loss (SURMOUNT-1). *It should be preferred* when weight burden is high, OSA, *T2D, and HF*.  · **Phentermine/Topiramate ER (full dose)**: 10.9% loss (EQUIP). *It May be considered* in patients needing rapid, potent weight loss. Milestone: >20% Weight Loss Required for reversal of advanced T2D, fibrosis, and OSA; matches surgical efficacy.  · **Tirzepatide 15 mg**: 20.9% loss (SURMOUNT-1); 62.8% AHI reduction in OSA (SURMOUNT-OSA). *Should be preferred* in severe obesity or obesity with OSA, MASLD, or severe insulin resistance.  · **Semaglutide 7.2 mg (investigational)**: 20.7% loss (STEP UP). *May be considered* for intensification when approved.  · **CagriSema (2.4/2.4 mg)**: 20.4% loss; ≥30% in 1 in 5 patients (REDEFINE 1). *Represents a highly effective future option*, not yet available. If Additional Weight Loss or Complication Reversal Is NeededBariatric Surgery Surgery offers the most potent and durable weight loss and **multisystem benefits**, including:  · **Cardiovascular Disease**: ↓ all-cause mortality (–29%), ↓ CV mortality (–53%) over 14.7 years (SOS). *It may be considered* in T2D or high CV risk with BMI ≥35.  · **Chronic Kidney Disease**: ↓ ESRD risk (HR = 0.27), improved eGFR and albuminuria. *It May be considered* when Obesity and CKD progresses despite medical therapy.  · **T2D & Prediabetes**: Up to 50% remission at 10 years; prevention of T2D in high-risk patients. *It should be preferred* for T2D with BMI ≥35 kg/m² or ≥30 kg/m² if uncontrolled.  · **T1D**: Weight loss 14–22%, insulin dose reduction sustained at 5 years. It *may be considered* in obesity with T1D to improve obesity and its related complications and reduce insulin requirements.  · **MASLD/MASH**: Steatosis resolved in 66%, ballooning 76%, fibrosis 40%; superior to drugs for MASH resolution (79% vs. 29%). *It Should be preferred* in advanced MASH, especially with fibrosis.  · **Osteoarthritis**: Pain and function improved in 65–77% at 3–7 years (LABS-2). It *should be preferred* in disabling OA where weight loss is a goal.  · **Obstructive Sleep Apnea**: ↓ AHI (–19.3 events/h), OSA remission in ~65% of cases. It *should be preferred* in moderate-severe OSA with obesity.  · **Cancer**: ↓ obesity-related cancer incidence by 32–33%; ↓ mortality by 48%. It **may be preferred** in younger people with high lifetime cancer risk. |
| --- |

| **Clinical Takeaways Box Pediatric and Adolescent Obesity Pharmacotherapy**  · **Lifestyle intervention remains first-line** therapy at all ages and should accompany any pharmacological treatment. Multidisciplinary support including nutritional, psychological, and behavioral care is essential to ensure efficacy, safety, and adherence.  · **GLP-1 RAs (liraglutide and semaglutide)** are now **first-line pharmacological options** in adolescents **≥12 years** (semaglutide) and **≥6 years** (liraglutide) with **moderate-to-severe obesity**, based on regulatory approvals and evidence of efficacy and safety.  o **Semaglutide 2.4 mg** may be preferred in patients with higher degrees of obesity and/or cardiometabolic risk. In STEP TEENS, it induced a **−16.1% reduction in BMI**, with 73% achieving ≥5% weight loss and improvements in cardiometabolic parameters (waist circumference, ALT, HbA1c).  o **Liraglutide 3.0 mg**, although less potent, may be considered for patients or settings where daily injection is acceptable. It demonstrated **moderate efficacy** and safety in children aged ≥6 years.  · **Phentermine/Topiramate ER** is FDA-approved from **age 12 years** in the US, and **may be considered** for adolescents with severe obesity when GLP-1 RAs are not tolerated or unavailable. It provided a **−10.4% to −8.1% BMI reduction** at 56 weeks. However, **EMA has not approved** its use due to potential neuropsychiatric and growth-related side effects. Caution and close monitoring are warranted.  · **Orlistat** may be considered from **age 12 years** (except in Canada), but is rarely used due to **limited efficacy** and high rates of gastrointestinal side effects. Its use should be restricted to motivated patients with appropriate dietary adherence.  · **Setmelanotide** is approved for **monogenic obesity** due to **biallelic POMC, PCSK1, LEPR deficiency** or **Bardet-Biedl syndrome** from **age 2 years**. It should be used only in **genetically confirmed cases** under specialist care.  · **Metreleptin** is approved from **age 2 years** for **congenital or acquired generalized lipodystrophy**, and may improve metabolic outcomes when initiated early. It does not have a role in common forms of pediatric obesity.  · **Metformin** is used **off-label** in adolescents with **insulin resistance or PCOS**, with modest weight effects. It may be considered when hyperinsulinemia is a dominant feature, but is not a weight-loss medication per se.  · **Age-specific considerations**:  o **0–6 years**: Pharmacotherapy is **not recommended** outside of monogenic or syndromic forms. Behavioral and family-based approaches remain the standard of care. Early-onset severe obesity with hyperphagia should prompt genetic evaluation.  o **6–12 years**: **Liraglutide (EU)** and **Setmelanotide (monogenic forms)** may be considered in selected patients. All other pharmacotherapies remain off-label.  o **12–18 years**: Adolescents with **severe obesity (BMI≥120% of the 95th percentile)** or **with obesity** **(BMI≥ 95th percentile)** **accompanied by complications** (e.g., prediabetes, PCOS, NAFLD, insulin resistance) **should be considered** for pharmacotherapy, particularly GLP-1 RAs.  · **Genetic testing should be considered** in children with:  o Obesity onset before age 5, especially <2 years  o Rapid weight gain (>2 SDs above norm)  o Hyperphagia  o Family history of early-onset severe obesity  o Dysmorphic features, developmental delay, or endocrinopathies (e.g., central hypothyroidism)  **All pharmacologic treatments should be prescribed** by or in consultation with **pediatric obesity specialists**, with structured **monitoring of growth, pubertal development, bone health, nutrition, and mental health.** |
| --- |

| **Clinical Takeaways Box: Obesity in Adults and Older People**  · **Pharmacologic therapy in adults should be phenotype-driven**, based on eating behavior, comorbidities (e.g., ASCVD, CKD, NAFLD), and patient priorities (e.g., diabetes prevention, fertility, function). In all cases, pharmacotherapy must be combined with structured lifestyle interventions and long-term follow-up. Adults (18–65 years): refers to the other boxes for adultsOlder Adults (≥65 years) · In older people, **treatment goals shift** toward preserving **mobility**, **function**, and **quality of life**, rather than targeting specific weight loss thresholds.  · Agents **should be selected based on tolerability**, risk of muscle loss, and cardiovascular safety. Avoid agents that may reduce appetite excessively or impair physical resilience.  · **Liraglutide 3.0 mg** may be preferred given **evidence from CVOTs** and **experience in elderly with T2D**, including weight reduction and cardiometabolic protection.  · **Orlistat** not recommended for GI side effects.  · **Naltrexone/bupropion** should generally be avoided due to risks of **CNS stimulation**, **insomnia**, **agitation**, and **blood pressure elevation**.  · **Phentermine** and other stimulants are **not recommended above age 75** due to **increased neuropsychiatric and cardiovascular risks**.  · **Caution is essential** in older adults to avoid **loss of lean mass**, **malnutrition**, and **adverse drug–drug interactions**, especially in patients with **frailty or polypharmacy**.  · **Individualized goals and shared decision-making** remain central to therapy success in both younger and older adults with obesity. |
| --- |

| **Clinical Takeaways Box Sarcopenic Obesity**  · **Sarcopenic obesity** is defined by the coexistence of excess fat mass and reduced skeletal muscle mass and/or strength. It is especially prevalent in **older adults** and people with **T2D**, **CKD**, and **CVD**, and is associated with increased risks of **frailty**, **falls**, **disability**, and **mortality**.  · Diagnosis is challenging: **BMI alone is insufficient**, as fat mass may mask progressive muscle loss. Screening with **SARC-F (score ≥4)** should prompt confirmatory assessment with **DXA**, **BIA**, **handgrip strength**, or **gait speed testing.**  · The **pathophysiology** involves **anabolic resistance**, **inflammation**, **insulin resistance**, **mitochondrial dysfunction**, and **physical inactivity**, leading to muscle catabolism despite caloric excess. Therapeutic Goals and Core Interventions · The primary aim is to **preserve or recover muscle mass and strength**, not just reduce weight.  · **Weight loss of 5–10%** may be beneficial if paired with improvements in **visceral adiposity**, **metabolic health**, and **function**. **Unsupervised or rapid weight loss** should be avoided in frail people due to the risk of excessive lean mass loss.  · **Nutritional strategies**:  o **Protein intake** ≥1.2 g/kg/day (up to 1.5 g/kg/day with energy restriction), **distributed across meals** to optimize anabolic signaling.  o **Vitamin D ≥800 IU/day** and **adequate calcium** are essential for muscle function and fall prevention.  · **Exercise**:  o **Progressive resistance training (2–3×/week)** is the most effective intervention to preserve muscle strength, type II fibers, and balance. Pharmacotherapy Considerations · **Liraglutide 3.0 mg daily**:  o Induces modest weight loss (~6%) while **preserving lean mass**, especially when combined with adequate **protein intake** and **exercise** .  o Favorable **cardiovascular profile** and **gradual kinetics** make it suitable for frail or elderly people.  · **Orlistat**:  o Offers ~3–4% weight loss without systemic exposure; ideal in **polymedicated patients** but requires **fat-soluble vitamin supplementation**.  · **Tirzepatide and Semaglutide**:  o Induce substantial weight loss (~15–24%), with **approximately two-thirds derived from adipose tissue**.  o Require **resistance training and nutritional support** to avoid functional decline during treatment  · **Naltrexone/bupropion**:  o Use with caution due to potential **neuropsychiatric side effects** (e.g., agitation, insomnia), particularly problematic in frail or cognitively impaired people.  · Effective management of sarcopenic obesity requires a **balanced strategy**: metabolic improvement should never come at the expense of **functional reserve**. Lifestyle measures remain the cornerstone, and **pharmacotherapy must be tailored** to support lean mass, physical performance, and nutritional adequacy. |
| --- |

| **Clinical Takeaways Box - Emotional Eating**  · **Emotional eating** involves food intake in response to **negative emotions** (e.g., anxiety, stress, sadness) rather than hunger. It is highly prevalent among people with obesity and is associated with **worse weight loss outcomes**, higher **relapse risk**, and greater susceptibility to **binge eating disorder**.  · The neurobiological basis includes **hyperactivity of cortico-limbic circuits** (amygdala, insula, orbitofrontal cortex), which can override satiety and promote **compulsive intake.**  · Successful pharmacotherapy in this phenotype may depend not only on weight reduction but also on the agent’s ability to modulate **reward sensitivity**, **craving**, and **disinhibition** Anti-Obesity Medications and Emotional Eating • **Naltrexone/Bupropion**: – Targets hypothalamic POMC neurons and mesolimbic reward pathways. In RCTs, it significantly improved control over eating, reduced food cravings, and enhanced the ability to resist urges to eat. Naltrexone/bupropion **should be preferred** in people with obesity and emotional eating, particularly when dysregulated reward and craving are dominant features. Caution is advised in patients with psychiatric comorbidities.  • **Semaglutide**: – Reduces emotional eating and food cue reactivity while increasing cognitive restraint via modulation of hypothalamic and mesolimbic circuits. Improves eating behavior and psychosocial well-being. Semaglutide **may be considered** in people with emotional eating, especially when weight loss and improved emotional regulation are desired outcomes.  • **Tirzepatide**: – In SURMOUNT-3, tirzepatide led to meaningful improvements in quality of life domains, including emotional well-being, among participants achieving ≥25% weight loss. Tirzepatide **may be considered** in people with obesity and emotional eating when significant weight reductions are required, although it does not directly modulate reward circuits.  • **Liraglutide**: – When added to intensive behavioral therapy, liraglutide enhanced short-term improvements in disinhibition and shape concern, although effects diminished by 1 year. Liraglutide **may be considered** as adjunct to structured behavioral interventions in emotional eating, particularly in early treatment phases.  • **Phentermine/Topiramate ER**: – Reduces binge-type and compulsive eating through appetite suppression and enhanced inhibitory control. Cognitive side effects may occur, particularly at higher doses. Phentermine/topiramate ER **may be considered** with caution in selected people with emotional eating, particularly when binge features are prominent and psychiatric stability is confirmed.  • **Orlistat**: – Does not act on central appetite or reward pathways and showed no added benefit in binge eating disorder. Orlistat is *not recommended* in people with emotional eating, as it lacks efficacy in modulating relevant behavioral drivers. |
| --- |

| Anti-Obesity Medications and Binge Eating Disorder (BED) • **Semaglutide**: – In prospective studies, semaglutide reduced binge frequency, food craving intensity, and improved scores on the Binge Eating Scale. Participants completing the Control of Eating Questionnaire experienced greater weight loss (–14.8% vs. –2.4%) and improved control over cravings, particularly for savory, sweet, and starchy foods. Semaglutide **may be considered** in people with BED and obesity when weight loss and craving control are therapeutic goals. Despite limited BED-specific RCTs, it may be beneficial through satiety and reward modulation.  • **Tirzepatide**: – Improved quality of life in SURMOUNT-3 participants with obesity; preclinical data suggest potential benefits in stress-related food seeking. A dedicated BED RCT (LIBERATE, NCT06847399) is planned. Tirzepatide **may be considered** in people with BED and obesity, particularly when substantial weight loss is prioritized. Dedicated clinical data are pending.  • **Liraglutide**: – In a 17-week RCT, liraglutide led to modest weight loss but showed no significant advantage over placebo in reducing binge episodes or achieving remission. Liraglutide **may be considered** in people with BED primarily for weight loss, but current evidence does not support its use for binge remission.  • **Naltrexone/Bupropion**: – Modulates dopaminergic and opioidergic pathways implicated in compulsive eating. In BED-specific trials, it did not significantly reduce binge frequency but led to greater weight loss and subjective improvement in severity. Naltrexone/bupropion **may be considered** in BED patients where emotional or addictive eating coexists with obesity but should not be relied upon to reduce binge episodes. Combination with psychotherapy should be considered.  • **Orlistat**: – In a 24-week RCT, orlistat led to greater weight loss and improved eating disorder symptoms compared to placebo. However, it does not target central mechanisms of binge eating and is often poorly tolerated. Orlistat **is not re**commended as first-line treatment in BED but may offer modest adjunctive benefit in weight-focused strategies, provided gastrointestinal side effects are managed. |
| --- |

| **Clinical Takeaways Box: Cancer Safety in Obesity Pharmacotherapy**   - **Obesity increases cancer risk**, particularly for endometrial, breast, colorectal, and pancreatic cancers. - **Weight loss reduces cancer incidence and mortality**, with bariatric surgery lowering obesity-related cancer risk by 32% - **GLP-1 RAs and thyroid cancer**: GLP-1 RAs were associated with a modest but significant increase in thyroid cancer risk in a 2025 meta-analysis of 50 RCTs (MH-OR 1.55, 95% CI 1.05–2.27), especially in longer-duration studies; no other malignancies showed significant associations. contraindicated in MTC or MEN2. - **No increased risk of pancreatic or GI cancers** has been found for GLP-1 RAs in SELECT or recent meta-analyses. - **Tirzepatide shows no elevated cancer risk** across major trials (>25,000 participants). - **No clear oncologic signals** have emerged for orlistat, phentermine/topiramate, or naltrexone/bupropion. - **Clinical focus**: AOMs appear safe from an oncologic perspective; long-term users should be monitored, especially when using incretin-based therapies. |
| --- |

| **Clinical Takeaways Box – Rare and Mechanism-Specific Obesity Syndromes**   - Partial lipodystrophies involve selective fat loss with severe metabolic complications; standard weight-loss strategies are often ineffective. - Tirzepatide improves glycemia, triglycerides, and steatosis in lipodystrophy, even without major fat mass changes. - Monogenic obesity (e.g., leptin deficiency) responds to targeted therapies like leptin or setmelanotide (POMC, LEPR, BBS, PCSK1 mutations). - Genetic testing is essential in early-onset obesity with hyperphagia or syndromic features to guide therapy. - Hypothalamic obesity, often post-tumoral, is challenging; setmelanotide and semaglutide show promise, especially with partial hypothalamic function preserved. - Treatment must be personalized, mechanism-based, and combined with structured lifestyle and psychological support. |
| --- |

| **Clinical Takeaways Box – AOM Use and Pregnancy**   - All AOMs are contraindicated in pregnancy due to teratogenicity, placental transfer, and lack of safety data. - Orlistat is not recommended despite minimal absorption, as it impairs micronutrient uptake. - GLP-1 RAs, tirzepatide, and naltrexone/bupropion are contraindicated due to fetal risk. - Phentermine/topiramate poses high teratogenic risk and is strictly avoided. - Long half-life agents (e.g., GLP-1 RAs, tirzepatide) must be stopped ≥1–2 months before conception. - In women with obesity planning pregnancy, short-term AOMs may reduce risk if paired with contraception, counseling, and timely discontinuation. |
| --- |

| **Anti-Obesity Medications and PCOS clinical takeaways**  **Semaglutide**: – In two small RCTs using 1.0 mg weekly over 16 weeks, semaglutide led to significantly greater weight loss than placebo (up to −7.8 kg, or −7.7% body weight). However, no trials have yet evaluated the 2.4 mg weight loss dose in PCOS. Semaglutide **may be considered** in women with PCOS and obesity who are candidates for pharmacologic weight loss. Larger studies at therapeutic doses are needed to confirm reproductive and metabolic effects.  • **Tirzepatide**: – No PCOS-specific trials are currently available. In the general population, tirzepatide 15 mg resulted in −20.9% weight loss at 72 weeks. Tirzepatide **may be considered** in women with PCOS and obesity when substantial weight loss is the primary therapeutic target. Dedicated studies are needed to assess reproductive and metabolic outcomes.  • **Liraglutide**: – In a 32-week RCT of adults with obesity and PCOS, liraglutide 3.0 mg daily led to −5.7% weight loss vs. −1.4% with placebo, with improvements in metabolic outcomes, free androgen index, and menstrual frequency. Liraglutide **should be preferred** in PCOS when weight loss and improved menstrual and metabolic parameters are desired. It may be especially useful when insulin resistance is prominent.  • **Orlistat**: – A 3-month RCT in women with obesity and PCOS showed orlistat improved weight loss (−6.4%), reduced waist-to-hip ratio, and improved total testosterone and lipid profiles, but had limited effect on other metabolic parameters. Orlistat **may be considered** in PCOS when systemic exposure is a concern and modest weight loss is acceptable. Gastrointestinal side effects and adherence should be monitored.  • **Naltrexone/Bupropion**: – No PCOS-specific data are available. Naltrexone/bupropion is not routinely recommended in PCOS due to lack of evidence and potential neuropsychiatric side effects. It may be reserved for cases with prominent emotional eating and preserved psychiatric stability.  • **Phentermine/Topiramate ER**: – In a small trial comparing phentermine/topiramate ER to exenatide, women with PCOS lost 8% body weight over 24 weeks, slightly exceeding weight loss reported in the general population. Phentermine/topiramate **may be considered** for short-term use in women with PCOS and severe obesity, but cardiovascular risk must be carefully assessed and monitored.  • **Metformin**: – Modest weight effects, with BMI reductions ~0.8 kg/m² in some trials; primarily used to improve insulin resistance, glucose and lipid profiles, and menstrual regularity. Metformin **is not recommended** for weight loss in PCOS but may be recommended in women with BMI ≥25 kg/m² to improve metabolic and reproductive outcomes. |
| --- |

| **Clinical Takeaways box – Cost-effectiveness of Obesity Treatments** • **Bariatric surgery** should be considered the most cost-effective obesity intervention, particularly in people with type 2 diabetes or severe obesity.  • **Orlistat** may be preferred in low-resource settings due to low cost and T2D prevention benefit, despite modest efficacy and GI side effects.  • **Semaglutide** offers QALY gains but may not be cost-effective at current pricing; value-based strategies or price reductions may improve accessibility.  • **Tirzepatide** provides the greatest health benefit but is not cost-effective at current cost; price reductions of ≥30% are needed to reach standard thresholds. |
| --- |

| **Clinical Takeaways Box – Non responder and combination therapies**  **Non-Response to AOMs:** Despite adherence, up to 15–17% of patients fail to achieve ≥5% weight loss in trials (e.g., STEP 1: 13.6%; SURMOUNT-1: 15% at 5 mg tirzepatide). Early weight loss,<0.5%/week in the first month,strongly predicts suboptimal response and may warrant treatment modification.   - **Combination Therapy in Refractory Obesity:** Rational polypharmacy is emerging for partial or non-responders:   - **GLP-1 RA + Naltrexone/Bupropion**: targets hedonic eating; effective in binge eating disorder, but requires close monitoring.   o **GLP-1 RA + Orlistat**: modest additional effect; potential option in cost-constrained settings.   - - **Semaglutide + Cagrilintide**: synergistic GLP-1 and amylin receptor agonism; up to 17% placebo-subtracted weight loss. - **Post-Bariatric Pharmacotherapy:** Up to 30% of patients experience inadequate weight loss or regain. AOMs are increasingly used to augment surgical outcomes:   - Semaglutide > liraglutide in post-operative weight regain.   - Tirzepatide improves both fat loss and lean mass preservation.   - Early liraglutide post-surgery may support durable outcomes in adherent patients. - **Sex-Based Differences in Response:**   - Women generally exhibit greater AOM-induced weight loss; possibly linked to hormonal, genetic (GLP-1R variants), and behavioral factors.   - Men are more prone to lean mass loss and benefit from resistance training and protein supplementation.   - In BS adverse events differ: women report more nausea and gallbladder issues; men have worse surgical outcomes and lower T2D remission. - **Toward Personalized Approaches:**   - Sex-specific algorithms (e.g., slower titration in women; muscle-preserving strategies in men) may enhance efficacy and tolerability.   - Future trials should stratify by sex and early treatment response to inform precision care in obesity management. |
| --- |

| Clinical Takeaways box – Safety and Monitoring in Anti-Obesity Pharmacotherapy • **GLP-1 RAs (semaglutide, liraglutide)** GLP-1 RAs should be considered safe and well tolerated. Gastrointestinal adverse events (nausea, vomiting, diarrhea) are common but typically transient. Liraglutide has demonstrated long-term cardiovascular and renal safety in T2D. **Monitoring should include**: renal function (especially with vomiting), gallbladder symptoms, and glycemic control in patients on glucose-lowering agents.  • **Dual Incretin Agonist (tirzepatide)** Tirzepatide may be preferred for patients requiring greater weight loss with GLP-1–like safety. GI events are dose-related. **Monitoring should include**: renal function (especially with vomiting), gallbladder symptoms, and glycemic control in patients on glucose-lowering agents.  • **Naltrexone/Bupropion** Naltrexone/bupropion should be avoided in patients with uncontrolled hypertension, seizure history, or opioid use. Psychiatric monitoring is essential due to neuropsychiatric risk. **Monitoring should include**: blood pressure, heart rate, mood symptoms, and substance use history. Annual CV risk assessment is advised.  • **Orlistat** Orlistat may be suitable in patients requiring a non-systemic option. Common adverse effects include GI disturbances and fat-soluble vitamin deficiency. **Monitoring should include**: liver enzymes, urinalysis, and vitamin levels in long-term users.  • **Phentermine/Topiramate ER** This combination may be effective for selected patients but should be avoided in people with recent CV events or psychiatric instability. It requires pregnancy prevention strategies due to topiramate teratogenicity. **Monitoring should include**: blood pressure, mood, cognition, and contraceptive adherence. |
| --- |

**SUPPLEMENTARY TABLE**

| **Supplementary Table 1. Effects of Off-Label Anti-Obesity Pharmacotherapy in Type 1 Diabetes: Glycemic, Insulin-Sparing, and Weight Outcomes** | | | | | | |
| --- | --- | --- | --- | --- | --- | --- |
| **Intervention** | **HbA1c Reduction** | **Insulin Dose Impact** | **Evidence Quality** | **Clinical Recommendation** | **Weight Loss** | **Evidence Source** |
| **Semaglutide** | −0.3% | −30% insulin dose | **High** | Consider off-label if insulin resistance and high metabolic burden with high insulin needs | −8.8 kg | [[50]](https://paperpile.com/c/herII6/JML2Q) |
| **Tirzepatide** | −0.9% | −31.6% insulin dose | **Low** | Consider off-label if insulin resistance and high metabolic burden with high insulin needs | −8.5% (median) | [[51]](https://paperpile.com/c/herII6/vPMpA) |
| This table summarizes data on the off-label use of anti-obesity medications in people with type 1 diabetes (T1D), focusing on their effects on HbA1c, insulin dose requirements, and body weight. The listed agents have been studied in small real-world cohorts of patients with T1D and obesity, typically in the context of marked insulin resistance and high metabolic burden. Evidence quality is classified as high or low based on the robustness of study design and replication. All treatments are off-label in T1D and should be considered only in selected patients, with close clinical monitoring. Weight loss is expressed as absolute (kg) or median percentage reduction where applicable. Abbreviations: T1D, type 1 diabetes; HbA1c, glycated hemoglobin. | | | | | | |
